# Contrasting effects of maize litter and litter-derived biochar on the temperature sensitivity of paddy soil organic matter decomposition

**DOI:** 10.3389/fmicb.2022.1008744

**Published:** 2022-09-29

**Authors:** Jun Cui, Tida Ge, Ming Nie, Yakov Kuzyakov, Sulaiman Alharbi, Changming Fang, Zifa Deng

**Affiliations:** ^1^School of Life Sciences, Nantong University, Nantong, China; ^2^Ministry of Education Key Laboratory for Biodiversity Science and Ecological Engineering, Department of Ecology and Evolutionary Biology, School of Life Sciences, Fudan University, Shanghai, China; ^3^State Key Laboratory for Managing Biotic and Chemical Threats to the Quality and Safety of Agro-products, Institute of Plant Virology, Ningbo University, Ningbo, China; ^4^Jiangsu Provincial Key Laboratory for Bioresources of Coastal Saline Soils, Jiangsu Coastal Biological Agriculture Synthetic Innovation Center, Yancheng Teachers’ University, Yancheng, China; ^5^Department of Agricultural Soil Science, Department of Soil Science of Temperate Ecosystems, University of Göttingen, Göttingen, Germany; ^6^Agro-Technological Institute, Peoples Friendship University of Russia (RUDN University), Moscow, Russia; ^7^Department of Botany and Microbiology, College of Science, King Saud University, Riyadh, Saudi Arabia

**Keywords:** priming effects, warming, three-source partitioning, enzyme Michaelis–Menten kinetics, phospholipid fatty acid, biochar

## Abstract

Organic matter input regulates the rate and temperature sensitivity (expressed as *Q*_10_) of soil organic matter (SOM) decomposition by changing microbial composition and activities. It remains unclear how the incorporation of litter-made biochar instead of litter affects the *Q*_10_ of SOM decomposition. Using a unique combination of two-and three-source partitioning methods (isotopic discrimination between C3/C4 pathways and ^14^C labeling), we investigated: (1) how maize litter versus litter-made biochar (of C4 origin) addition influenced the *Q*_10_ of SOM (C3 origin) under 10°C warming, and (2) how the litter or biochar amendments affected the *Q*_10_ of ^14^C-labeled fresh organic matter (FOM) after long-term incubation. Compared with biochar addition, litter increased the rates and *Q*_10_ of mass-specific respiration, SOM and FOM decomposition, as well as the contents of SOM-derived dissolved organic C (DOC) and total phospholipid fatty acids (PLFA). Litter-amended soils have much higher activities (*V*_max_) of β-glucosidase, N-acetyl-β-glucosaminidase, and leucine aminopeptidase, suggesting larger enzyme pools than in soils with biochar. The *Q*_10_ of enzyme *V*_max_ (1.6–2.0) and *K*_m_ (1.2–1.4) were similar between litter-and biochar-amended soils, and remained stable with warming. However, warming reduced microbial biomass (PLFA) and enzyme activity (*V*_max_), suggesting decreased enzyme production associated with smaller microbial biomass or faster enzyme turnover at higher temperatures. Reductions in PLFA content and enzyme *V*_max_ due to warming were larger in litter-amended soils (by 31%) than in the control and biochar-amended soils (by 4–11%), implying the active litter-feeding microorganisms have a smaller degree of heat tolerance than the inactive microorganisms under biochar amendments. The reduction in enzyme activity (*V*_max_) by warming was lower in soils with biochar than in the control soil. Our modeling suggested that the higher *Q*_10_ in litter-amended soils was mainly caused by faster C loss under warming, linked to reductions in microbial biomass and growth efficiency, rather than the slightly increased SOM-originated substrate availability (DOC). Overall, using straw-made biochar instead of straw *per se* as a soil amendment lowers the *Q*_10_ of SOM and FOM by making microbial communities and enzyme pools more temperature-tolerant, and consequently reduces SOM losses under warming.

## Introduction

Climate warming, concomitant with rising atmospheric CO_2_ concentration, is projected to elevate the Earth’s temperature by 1.5–3.5°C by 2100 (IPCC, 2022). It is highly uncertain whether warming will accelerate the transfer of the enormous global soil C stock to the atmosphere, which implies a positive feedback between climate and the terrestrial C cycle (Davidson and Janssens, 2006). Estimation of the temperature sensitivity of soil organic matter (SOM) decomposition (usually expressed as *Q*_10_, the factor by which the decomposition rate increases with a 10°C temperature rise) is therefore critical to future climate projections (Jones et al., 2005; Todd-Brown et al., 2013). The *Q*_10_ of SOM decomposition is partly determined by substrate availability (Gershenson et al., 2009; Pang et al., 2015), which in turn is controlled by soil C stabilization mechanisms (Conant et al., 2011). Further, *Q*_10_ is tightly linked to microbial decomposer characteristics, such as C use efficiency (CUE) and extracellular enzyme kinetics (Allison et al., 2010; Bradford, 2013).

A major microbial regulation over SOM decomposition is through catalysis by extracellular enzymes, a rate-limiting step of decomposition generally modeled as temperature-dependent Michaelis–Menten kinetics (Davidson et al., 2006, 2011):


$$V=\frac{V_{\max}\times \left[S\right]}{K_{m}+\left[S\right]}$$

where *V* is the decomposition rate of the substrate, [*S*] is the substrate concentration in the soil solution or solid phase, *V*_max_ is the maximum rate of the enzyme-catalyzed reaction, and *K*_m_ is the half-saturation constant (the substrate concentration at which *V* equals half *V*_max_) which is indicative of substrate-enzyme affinity. *V*_max_ and *K*_m_ are both intrinsically temperature-sensitive, and their relative changes with temperature determine the apparent temperature sensitivity of reaction rates, which is particularly important at low [*S*] (Razavi et al., 2015). *V*_max_, *K*_m_ and their *Q*_10_ are crucial parameters in new-generation soil biogeochemical models that link microbial physiology to C processes (Allison, 2012; Wieder et al., 2014). Decreases in *V*_max_ or increases in *K*_m_ with warming may contribute to microbial thermal acclimation by warming (Allison et al., 2018).

Fresh C supply stimulates microorganisms to secrete enzymes and thereby promote SOM decomposition, which is termed the “priming effect” (Kuzyakov, 2010). Moreover, the temperature sensitivity of soil C mineralization (either of SOM or fresh C input) could be increased by new substrate inputs (Zhu and Cheng, 2011), whereas substrate shortage tends to have the opposite effect (Moinet et al., 2018; Su et al., 2022). This was attributed to the positive correlation between *Q*_10_ and the item [*S*] (i.e., the substrate concentration) in the Michaelis–Menten equation, because the effects of increasing *V*_max_ with temperature are more strongly counterbalanced by increasing *K*_m_ at lower [*S*] (Davidson et al., 2006). However, the possible microbiological mechanisms underlying the changed *Q*_10_ under exogenous substrate inputs, such as the temperature responses of soil enzyme kinetics (*V*_max_ and *K*_m_) and microbial physiology (e.g., CUE and microbial turnover), have rarely been considered. In addition, few studies have disentangled the temperature sensitivity of SOM and newly added fresh substrates (Zhu and Cheng, 2011; Wei et al., 2021), which should behave differently under climate warming given their distinct decomposability (Davidson and Janssens, 2006).

Converting plant biomass (tree, grass, or crop residues) into biochar by pyrolysis, and applying biochar to the soil, is a measure of abating climate change by C sequestration (Lehmann, 2007; Woolf et al., 2010). This is primarily based on the chemical inertness of biochar and its very long residence time in the soil (hundreds to thousands of years; Kuzyakov et al., 2014), especially when compared with the rapid decomposition of plant litter. It should be noted that plant biomass pyrolysis to biochar deprives soil organisms of a substantial amount of labile C, which would normally return to the soil under natural conditions, thereby profoundly affecting ecosystem processes. Many studies have compared the effects of litter and litter-derived biochar on greenhouse gas emissions, N cycling, enzyme activities, and microbial C utilization (Wu et al., 2013; Shen et al., 2014; Liu et al., 2020). However, it has not been considered that converting litter to biochar, which decreases labile C inputs to the soil, may lower the temperature sensitivity of SOM decomposition. This is because a lack of utilizable C reduces the growth of microbial biomass and extracellular enzyme production, lowering the depolymerization of SOM (and hence SOM-derived substrates, [*S*]). In addition, microbial communities with greater growing biomass are more temperature-sensitive (Larionova et al., 2007). This should result in a higher *Q*_10_ of SOM decomposition under litter than under biochar amendment (Thiessen et al., 2013). On the other hand, biochar may reduce the temperature responses of SOM mineralization by lowering microbial activities (e.g., the metabolic quotient; Zhang et al., 2022). To date, however, the effects of litter and litter-derived biochar on the temperature sensitivity of soil C decomposition have not been assessed.

The goals of this study were (1) to compare the effects of litter and litter-derived biochar on the *Q*_10_ of the decomposition of SOM and freshly added organic substances, and (2) to investigate the underlying mechanisms from the perspective of enzyme kinetics, microbial physiology, and substrate availability. Soils amended with maize litter or litter-derived biochar were subjected to 10°C warming at the early and late stages of a long-term incubation. The *Q*_10_ of SOM decomposition can be distinguished from that of biochar or litter based on the distinct isotopic signatures of C4 (maize) and C3 (SOM) materials. After long-term incubation, we applied a secondary addition of ^14^C-labeled wheat litter to all soils to assess how prior amendments affected the *Q*_10_ of fresh C inputs. The temperature responses of Michaelis–Menten kinetics of soil enzymes, microbial phospholipid fatty acid (PLFA) profiles, and soil substrate availability (dissolved organic matter) for microorganisms were analyzed to elucidate the mechanisms responsible for the *Q*_10_ of organic matter decomposition. The unique combination of isotopic approaches, with analyses of PLFA biomarkers and enzyme kinetics, provides useful information about how the lability of amendments influences soil C feedbacks to warmer climates.

## Materials and methods

### Soil collection and biochar production

Soil was collected from the plow horizon (Ap horizon, 0–10 cm) of an old paddy rice field located in northern Jiangsu Province, China. The region is characterized by a typical subtropical climate, with an annual precipitation of 1,000 mm and an average temperature of 14°C. The soil had a silty texture (silt: 88%; clay: 3.5%) and could be tentatively classified as Anthrosol (WRB, 2015). Soils from ten points in the field were homogenized by passing through a 2-mm sieve, and handpicked to remove plant residues and stones prior to incubation. The basic soil properties are listed in Table 1.

**Table 1:** Basic properties of the soil and amendments of maize litter and biochar.

	Soil	Maize litter	Biochar (400°C)	Biochar (650°C)
Total C (%)	1.95 ± 0.03	45.4 ± 0.05	49.1 ± 0.09	58.1 ± 0.17
Total N (%)	0.19 ± 0.001	1.41 ± 0.01	2.54 ± 0.02	1.9 ± 0.03
C:N ratio	10.2 ± 0.01	32.3 ± 0.22	19.3 ± 0.10	30.6 ± 0.53
pH	6.82 ± 0.10	ND	8.69 ± 0.01	9.30 ± 0.01
DOC (mg/g)	0.05 ± 0.004	ND	1.31 ± 0.25	1.53 ± 0.24
δ^13^C (‰)	−27.3 ± 0.21	−12.3 ± 0.33	−12.07 ± 0.13	−12.23 ± 0.04

Values are means ± standard errors (*n* = 3).

Biochar was prepared from maize litter (leaves) at 400°C and 650°C. Finely ball-milled maize litter was passed through a 2-mm sieve and tightly filled into a ceramic crucible (iØ/oØ = 46/50 mm × 40 mm high) prior to pyrolysis in a muffle furnace. The temperature of the muffle furnace was slowly raised from room temperature to 400 or 650°C at a rate of 4.2°C min^−1^ and kept at the set temperature for 4 h. The charring process yielded biochar with a mass equivalent to 30 and 15% of the initial litter mass at 400°C and 650°C, respectively. The biochar was milled and 0.5 mm-sieved before being added to the soil. The basic properties of biochar are listed in Table 1.

### Experimental layout and soil incubation procedures

Long-term soil incubation was conducted with four treatments, i.e., soils with no amendment (Control), soils amended with maize litter (Litter), biochar produced at 400°C (BC400), and biochar produced at 650°C (BC650). The maize litter was added at a rate of 30 mg g^−1^ soil (o.d. basis), while the rates of biochar addition were 30 and 15% (the yield rate of biochar) of litter addition rate for BC400 (9.2 mg g^−1^ soil) and BC650 (5.6 mg g^−1^ soil), respectively. Biochar was amended at such rates so that the litter mass required to produce the added biochar was equivalent to the added maize litter in the litter treatment. All soils were adjusted to 50% water-holding capacity and incubated at 20°C in 150 ml glass flasks, which were loosely capped, and distilled water was added periodically to maintain constant soil moisture.

**Figure 1:**
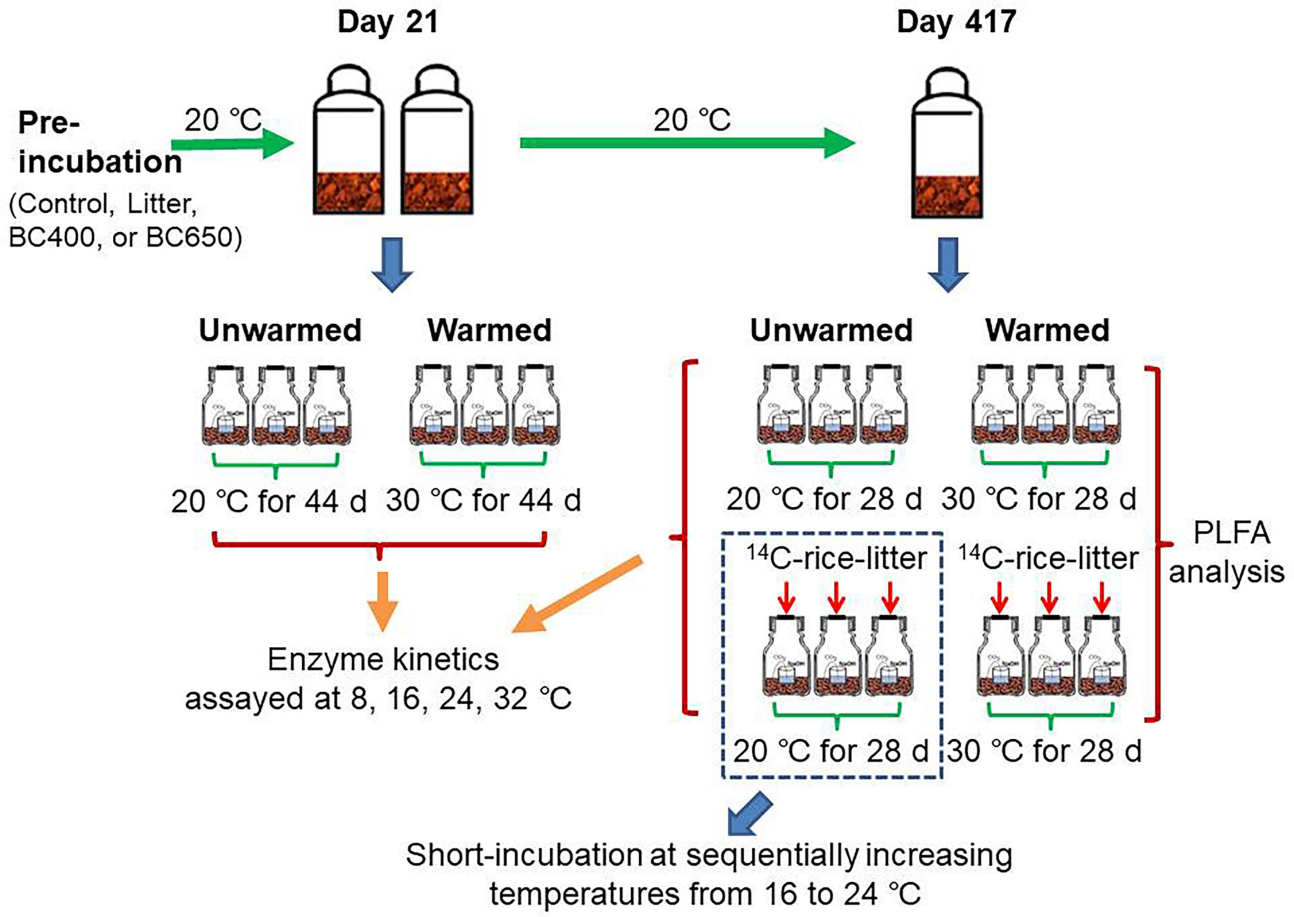
Layout of long-term incubation and warming experiments. Briefly, soils without/with litter or biochar addition were incubated at 20°C for 21 and 417 days before being incubated in 1.2 L glass jars with NaOH traps for a short period at 20°C (unwarmed) and 30°C (warmed). Part of soils at day 417 received secondary amendments of ^14^C-labeled rice litter prior to the warming. Additional incubation at sequentially increasing temperatures was conducted for soils that had received secondary ^14^C-labeled litter addition at day 417 and had been subsequently incubated in glass jars at 20°C for 48 days.

On day 21 of the incubation, soils of 18 g dry weight were transferred into small plastic vials and placed into airtight 1.2-L jars, together with another vial containing 15 ml 1 M NaOH solution. Thereafter, three replicates per treatment were maintained at 20°C, while the other three were incubated at 30°C for 44 days to mimic a short-term soil warming event. Such magnitude of temperature rise was larger than those estimated in various climate change projections (IPCC, 2022), but was generally adopted in incubation studies to maximize temperature effects over short time scales (e.g., Fang et al., 2014; Xu et al., 2022). Soil CO_2_ emitted over the study period, as well as its isotopic composition, was determined after 44 days of warming (Figure 1).

Short-term warming was also performed after 417 days of long-term incubation, but additional treatments were set up with the secondary addition of dried ^14^C-labeled rice leaves. Rice litter instead of maize litter was used because crop rotations commonly occur in paddy fields of the study area. A portion of soils (7 g on a dry weight basis) keeping the original treatments (Control, Litter, BC400, or BC650) were treated as above, that is, incubated at 20°C and 30°C with NaOH vials. The remaining soils received a secondary incorporation of ^14^C-labeled rice leaves at a rate of 30 mg g^−1^, in addition to their original amendments, and were then placed at 20°C and 30°C for short-term warming. The ^14^C-labeling procedures for rice have been described by Ge et al. (2012). Briefly, rice seedlings of roughly 0.1 g dry weight were transplanted and grown in an air-tight chamber for continuous labeling with ^14^CO_2_ generated from Na_2_^14^CO_3_ for >2 months, after which their leaves were harvested. The warming lasted 28 days, following which soil CO_2_ emissions, its ^13^Csignatures, and ^14^C activities were analyzed.

To further investigate *Q*_10_ of the freshly added organic matter, an incubation regime using sequentially changing temperatures was adopted for soils receiving the secondary litter amendment after short-term warming was completed. Briefly, the incubation temperature was slowly decreased from 20°C to 16°C at a rate of 1°C every 6 h, and then increased from 16, 18, 20, 22, to 24°C at a step of 2°C every 3–9 days. The duration at each temperature depends on the specific CO_2_ emission rate. The NaOH solution used for CO_2_ trapping was collected and replenished at the end of the incubation at each temperature. The trapped CO_2_ and its ^14^C signal were analyzed later.

### Chemical and isotopic analysis

For soils undergoing short-term warming on days 21 and 417, the amount and ^13^C or ^14^C signatures of NaOH-trapped CO_2_ were determined using the following procedure. First, if ^14^C activity was analyzed, a 5 ml aliquot was removed from the collected 15 ml NaOH solution and stored until ^14^C measurement on a scintillation counter (LS-6500, Beckman, Germany). The remaining NaOH solution was precipitated with excess 1 M SrCl_2_ and titrated with 0.5 M HCl to quantify the trapped CO_2_. The precipitate (SrCO_3_) was washed with 50 ml deionized water by centrifuging at 9,000 rpm and discarding the supernatants, which was repeated three times. Finally, the SrCO_3_ precipitate was dried at 50°C for ^13^C analysis using a MAT 253 isotope ratio mass spectrometer (IRMS) equipped with a Kiel IV Carbonate Device (Thermo Scientific, United States; precision: ±0.04‰).

Soil pH was measured in deionized water extracts at a soil:water ratio of 1:5 using a pH electrode (Mettler Toledo FE28, Switzerland), whereas the pH of the biochar was measured at a biochar:water ratio of 1:15. To determine the dissolved organic C (DOC) content, soils or biochar were extracted with 0.05 M K_2_SO_4_ at a soil:K_2_SO_4_ ratio of 1:4 or biochar:K_2_SO_4_ ratio of 1:10. The total organic C of the K_2_SO_4_ extracts was analyzed using a TOC analyzer (Multi N/C 2100, Analytik Jena, Germany). The total C and N, as well as the ^13^C composition, of the soil/biochar solids were determined using an elemental analyzer (Vario Macro Cube, Elementar, Germany) coupled to an IRMS (MAT 253, Thermo Finnigan, United States; precision: ±0.10‰).

### Enzyme assays

Unwarmed and warmed soils at both early and late incubation stages were analyzed for enzyme kinetics to reflect how different amendments affected the temperature sensitivities of enzyme *V*_max_ and *K*_m_ (Figure 1). The kinetics of three enzymes targeting soil C-and N-containing substrates, β-glucosidase (BG; EC: 3.2.1.21), N-acetyl-β-glucosaminidase (NAG; EC: 3.2.1.14), and leucine aminopeptidase (LAP; EC: 3.4.11.1), were analyzed at different temperatures. Enzyme assays were performed following the method described by Allison et al. (2018). Briefly, homogenous soil slurries were prepared by dispersing 3 g of moist soil in 120 ml buffer. The buffer contained 14 g L^−1^ citric acid, 6.3 g L^−1^ boric acid, 12.1 g L^−1^ Trizma base, 11.6 g L^−1^ maleic acid, and 19.5 g L^−1^ NaOH, and were adjusted to the same pH (6.8) with soil. Thereafter, 300 μl soil homogenate was combined with75 μl of substrates in each well of a 96-well microplate, which was incubated at 8, 16, 24, and 32°C for 4 h (but 2 h for BG and LAP on day 21). All enzyme activities were assayed for Michaelis–Menten kinetics, with seven substrate concentrations spanning the range of 10–600 μM.

### Phospholipid fatty acid analysis

The extraction and analysis of PLFAs followed the procedures described by Ge et al. (2017). Fatty acids were extracted from 3 g of freeze-dried samples in 15.2 ml of chloroform:methanol:citrate (1:2:0.8) buffer. Phospholipids in the extracts were separated from neutral lipids and glycolipids by using a silica-bonded phase column (SPE-Si, Supelco, Poole, UK). Subsequently, the phospholipids were methylated to fatty acid methyl esters (FAMEs), which were quantified using a gas chromatograph (N6890, Agilent, USA) and identified using the MIDI Sherlock Microbial Identification System 4.5 (Newark, DE, USA). The 19:0 methyl ester was used as an internal standard. PLFA analysis was only conducted for warmed and unwarmed soils at the late incubation stage, to examine how different microbial groups responded to the 10°C warming and whether this was changed by secondary litter addition (Figure 1).

PLFA markers for various microbial groups are listed in Supplementary Table S1, with monounsaturated fatty acids used as indicators for gram-negative bacteria, iso-and anteiso-branched fatty acids for gram-positive bacteria, 10-methyl fatty acids for actinomycetes, and 18:2ω6c and 18:1ω9c for fungi (Zhang et al., 2017). Two calculated indicators, the ratio of two cyclopropyl fatty acids (cy17:0 and cy19:0) to their precursors (Cy/Pre), and the degree of PLFA unsaturation, were used to reflect microbial responses to temperature stress. The PLFA unsaturation was calculated as follows:


(1) $$\mathrm{Unsaturation}=\sum \frac{\left[PLFA_{\mathrm{unsat}}\right]\times N_{db}}{\mathrm{total PLFA}}\times 100\%$$

where [*PLFA*_unsat_] refers to the concentration of a specific unsaturated PLFA in the sample, and *N*_*db*_ is the number of double bonds in the PLFA.

### Statistical analyses

The stable C isotopic composition of samples is expressed as *δ*^13^C values defined by:


(2) $$\delta^{13}C\;\left(‰\right)=\left[\left(R_{\mathrm{sample}}/R_{V-\mathrm{PDB}}\right)-1\right]\times 1,000$$

where *R*_sample_ and *R*_v-PDB_ are the ^13^C/^12^C ratios in the samples and Vienna Pee Dee Belemnite (V-PDB) standard, respectively. When no ^14^C-labeled rice litter was involved, the contributions of maize-originating litter/biochar and native SOM to soil CO_2_ emission was calculated using a simple two-source mixing model:


(3) $$C_{L}=C_{t}\times \frac{\delta^{13}C_{t}-\delta^{13}C_{\mathrm{SOM}}}{\delta^{13}C_{L}-\delta^{13}C_{\mathrm{SOM}}}$$

(4) $$C_{\mathrm{SOM}}=C_{t}-C_{L}$$

where *C_L_*, *C*_SOM_, and *C_t_* are the C from maize litter, native SOM, and bulk soil (mg kg^−1^), respectively; *δ*^13^*C_L_*, *δ*^13^*C*_SOM_, and *δ*^13^*C_t_* refer to the *δ*^13^C values (‰) of maize litter (−12.32 ± 0.33‰), SOM (−27.33 ± 0.21‰), and total soil C, respectively.

When both maize-originating litter/biochar and ^14^C-labeled rice litter were present in the soil, the total CO_2_ released from the soil was partitioned into three C sources, i.e., the maize-derived litter or biochar, the rice litter, and native SOM, using the approach of Blagodatskaya et al. (2011). In the first step, the contributions of ^14^C-labeled rice litter and other non-rice C sources were calculated based on their specific ^14^C activity.


(5) $$C_{\mathrm{rice}}=\frac{\left(DPM_{s}-DPM_{bl}\right)\times V_{\mathrm{NaOH}}}{{}^{14}C_{\mathrm{RL}}/{\left[C\right]}_{\mathrm{RL}}}$$

(6) $$C_{\mathrm{non}-\mathrm{rice}}=C_{\mathrm{bulk}}-C_{\mathrm{rice}}$$

where *C*_rice_ (mg C), *C*_non-rice_ (mg C), and *C*_bulk_ (mg C) are C derived from rice, non-rice sources (maize and native SOM), and bulk soil, respectively; *DPM_s_* and *DPM_bl_* are the ^14^C activity (decay per minute, DPM ml^−1^) of the NaOH solution for samples and the blank, respectively; ^14^*C*_RL_ (DPM g^−1^) and [*C*]_RL_ (mg C g^−1^) are the specific ^14^C activity and C content of the rice litter, respectively; and *V*_NaOH_ is the volume (ml) of NaOH for CO_2_ trapping. In the second step, C from non-rice sources was partitioned into native SOM and maize-originating C (litter or biochar), according to the following equations:

(7) $$\delta^{13}C_{\mathrm{non}-\mathrm{rice}}=\frac{C_{t}\times \delta^{13}C_{t}-C_{\mathrm{rice}}\times \delta^{13}C_{\mathrm{rice}}}{C_{\mathrm{non}-\mathrm{rice}}}$$

(8) $$C_{\mathrm{maize}}=C_{\mathrm{non}-\mathrm{rice}}\times \frac{\delta^{13}C_{\mathrm{non}-\mathrm{rice}}-\delta^{13}C_{\mathrm{SOM}}}{\delta^{13}C_{\mathrm{maize}}-\delta^{13}C_{\mathrm{SOM}}}$$

(9) $$C_{\mathrm{SOM}}=C_{\mathrm{non}-\mathrm{rice}}-C_{\mathrm{maize}}$$

where *C*_rice_ (*δ*^13^*C*_rice_), *C*_non-rice_ (*δ*^13^*C*_non-rice_), *C*_maize_ (*δ*^13^*C*_maize_), *C*_SOM_ (*δ*^13^*C*_SOM_), and *C*_t_ (*δ*^13^C_t_) refer to C (*δ*^13^Cvalues) from rice (*δ*^13^C_rice_: −25.75 ± 0.22‰), non-rice sources, maize (litter or biochar), native SOM, and bulk soil, respectively. The *Q*_10_ for the decomposition of total soil C and specific C pools was calculated as the ratio of their mineralization rates at 30°C to those at 20°C.

The kinetic parameters for enzymes, half-saturation constant (*K*_m_), and maximal velocity (*V*_max_) at each assay temperature were derived by fitting soil enzyme activities at increasing substrate concentrations to the Michaelis–Menten equation. Fitting was performed using the nonlinear least squares (NLS) function in R 4.1.0. *Q*_10_ for *V*_max_ and *K*_m_ was calculated using the following equation:


(10) $$Q_{10}={\left(\frac{V_{\max32}}{V_{\max8}}\right)}^{\frac{10}{24\;}}\;\mathrm{or}\;{\left(\frac{K_{m32}}{K_{m8}}\right)}^{\frac{10}{24\;}}$$

where *V*_max32_ (*K*_m32_) and *V*_max8_ (*K*_m8_) are the fitted *V*_max_ (*K*_m_) values at the assay temperatures of 32 and 8°C, respectively. The relationships between *V*_max_ or *K*_m_ and assay temperature were exponential (except for *K*_m_ of LAP on day 417), and *V*_max_ and *K*_m_ were log-transformed when plotting them versus assay temperature. Mass-specific respiration was expressed as CO_2_ emitted per unit PLFA content over a certain incubation period.

One-way analysis of variance (ANOVA) was used to test the effects of amendments on soil C, *Q*_10_ and PLFA contents, followed by Duncan’s post-hoc test. For the changing-temperature incubation, an one-way repeated-measures ANOVA was conducted to test the between-treatment differences in the decomposition rates of ^14^C-labeled rice litter. Principal component analysis (PCA) was applied for ordination of the PLFA composition of the soil samples using PC-ORD 5 (MjM Software, United States).

### Modeling analysis of variables determining *Q*_10_ of SOM mineralization

We constructed a simple modeling analysis of the roles of enzyme kinetics (*V*_max_ and *K*_m_), soil substrate availability (using DOC as a proxy), and microbial physiological variables (microbial turnover) in determining *Q*_10_ of SOM mineralization over a short period of warming after 417 days of incubation. Following previous studies (Allison et al., 2010), we assumed SOM mineralization to be a Michaelis–Menten process affected by microbial CUE:


(11) $$R\left(T\right)=\frac{V_{\max-T0}\times Q_{10-\mathrm{vmax}}{}^{\left(T-T0\right)/10}\times \left[S\right]}{K_{m-T0}\times Q_{10-\mathrm{km}}{}^{\left(T-T0\right)/10}+\left[S\right]}\times \left(1-\mathrm{CUE}\right)$$

where *R*(T) is the temperature-dependent soil respiration rate originating from SOM at a given time point, *V*_max-T0_ (*K*_m-T0_) is *V*_max_ (*K*_m_) at a reference temperature T0, *Q*_10-vmax_ (*Q*_10-km_) is the intrinsic *Q*_10_ for enzyme *V*_max_ (*K*_m_), and [*S*] is the SOM-derived substrate content at the incubation temperature T. Based on soil incubation data at 20°C and 30°C, the instantaneous *Q*_10i_ of SOM mineralization at any time point can be derived as


(12) $$Q_{10i}=\frac{R\left(30\right)}{R\left(20\right)}=\frac{D_{f}\times Q_{10-\mathrm{vmax}}\times \left(1+{\left[S\right]}_{20}/K_{m20}\right)}{Q_{10-Km}/Q_{10\left[S\right]}+{\left[S\right]}_{20}/K_{m20}}\times Q_{10-R\%}$$

(13) $$D_{f}=\frac{V_{\max}\left(30\right)}{V_{\max}\left(20\right)}$$

(14) $$Q_{10-R\%}=\frac{1-CUE_{30}}{1-CUE_{20}}=\frac{1-D_{f}\times CUE_{20}}{1-CUE_{20}}$$

where [*S*]_20_/*K*_m20_ is the ratio of substrate content ([*S*]) to *K*_m_ at 20°C, *Q*_10[S]_ is the ratio of SOM-derived substrate content at 30°C to that at 20°C, *CUE*_20(30)_ is CUE at 20 (30) °C, *D_f_* is the decay factor by which enzyme pools (indicated by *V*_max_ and linked to microbial biomass) were decreased due to soil warming, and *Q*_10-R%_ is the temperature sensitivity of the proportion of microbial assimilated C loss *via* cell respiration (i.e., C that is not ultimately used for biomass construction) as a function of CUE at different temperatures. We only considered *Q*_10i_ at the late incubation stage in the absence of secondarily added rice litter, because in this case, soil respiration before warming should have reached a near-equilibrium state, and thus *Q*_10i_ could be easily linked to the temperature sensitivity of cumulative SOM mineralization (*Q*_10t_):


(15) $$Q_{10t}=\overset{t}{\underset{i=1}{\sum }}\frac{Q_{10i}\times R_{20}}{t\times R_{20}}=\overset{t}{\underset{i=1}{\sum }}Q_{10i}/t$$

where *Q*_10i_ corresponds to a short time interval (an hour) of the warming period *t* and *R*_20_ is the SOM mineralization at 20°C, which is assumed to be invariant with time.

The ratio of DOC from soils incubated at 30°C to that incubated at 20°C was used to approximate *Q*_10[S]_ (Tables 2, 3). *Q*_10-vmax_ and *Q*_10-Km_ in Equations (11, 12) were parameterized with measured values for BG at 20°C (Table 3), considering that BG enzymes catalyze the hydrolysis of cellobiose and other organic substrates, and their kinetics should be highly correlated with that for overall SOM mineralization. However, *Q*_10-R%_ had to be estimated based on previously reported CUE values in the literature. [*S*]_20_ was estimated by fitting the measured BG activities at varying substrate concentrations to a modified Michaelis–Menten equation (Larionova et al., 2007):


(16) $$V=\frac{V_{\max}\times \left(\left[C\right]+{\left[S\right]}_{20}\right)}{K_{m}+\left[C\right]+{\left[S\right]}_{20}}$$

where *V* is BG activity at the exogenous substrate concentration of [C] at 20°C, *V*_max_ is the maximum reaction velocity of BG, *K*_m_ is the half-saturation constant, and [*S*]_20_ is the concentration of substrates derived from native SOM at 20°C.

**Table 2:** Dissolved organic C (DOC), total phospholipid fatty acids (PLFA), and mass-specific respiration (*R*_mass_) at the late incubate stage.

Treatments	DOC (mg C kg^−1^)^a^			Total PLFA (nmol g^−1^)			*R*_mass_ (μg C nmol^−1^ PLFA d^−1^)^b^		
	20°C	30°C	*R*_30/20_	20°C	30°C	*R*_30/20_	20°C	30°C	*R*_30/20_
− ^14^C rice litter									
Control	56 ± 2.7b	53 ± 6.18b	0.94 ± 0.03a	38.0 ± 1.8a	31.1 ± 2a	0.82 ± 0.05a	0.42 ± 0.05a	0.42 ± 0.03b	0.99 ± 0.08b
Litter	77 ± 35a	91 ± 21.99a	1.1 ± 0.1a	57.3 ± 4a	34.0 ± 4.2a	0.59 ± 0.07b	0.34 ± 0.02a	0.78 ± 0.08a	2.29 ± 0.24a
BC400	55 ± 8.6b	54 ± 2.28b	0.99 ± 0.01a	34.3 ± 3.1a	31.2 ± 1.3a	0.91 ± 0.04a	0.45 ± 0.11a	0.48 ± 0.06b	1.07 ± 0.13b
BC650	54 ± 5b	53 ± 17.08b	0.99 ± 0.18a	40.3 ± 11a	32.8 ± 2a	0.94 ± 0.06a	0.53 ± 0.08a	0.40 ± 0.03b	0.74 ± 0.06b
+ ^14^C rice litter									
Control	85 ± 49a	85 ± 37a	0.87 ± 0.15a	71.0 ± 0.2a	44.7 ± 2.3a	0.63 ± 0.03a	1.34 ± 0.02b	2.22 ± 0.04bc	1.66 ± 0.03d
Litter	86 ± 27a	110 ± 2.1a	1.27 ± 0.01a	56.4 ± 3.5a	33.2 ± 1.7a	0.59 ± 0.03a	1.54 ± 0.04a	3.49 ± 0.07a	2.27 ± 0.05b
BC400	70 ± 16a	76 ± 58a	1.18 ± 0.2a	77.2 ± 10a	43.0 ± 1.8a	0.56 ± 0.02a	1.04 ± 0.02c	2.2 ± 0.03c	2.12 ± 0.03c
BC650	93 ± 27a	90 ± 5.9a	1.14 ± 0.02a	87.0 ± 3.8a	38.3 ± 7.1a	0.44 ± 0.08a	0.85 ± 0.01d	2.35 ± 0.02b	2.75 ± 0.02a

*R*_30/20_ is the ratio of DOC or PLFA at 30°C–20°C. For each temperature with or without secondary ^14^C litter addition, lowercase letters in a column indicate significant differences between treatments. Values are presented as mean ± standard error (*n* = 3).

a DOC in litter-and biochar-amended soils without ^14^C-litter addition almost entirely originated from SOM as indicated by ^13^C signatures (not shown for clarity) of DOC.

b Respiration was averaged over 28 days of incubation to calculated *R*_mass_.

**Table 3:** Parameters used to simulate temporal changes in the instantaneous (*Q*_10i_) and cumulative (*Q*_10t_) temperature sensitivity of SOM mineralization depending on soil amendments.

Parameter	Units	Control	Litter	BC400 &BC650
*D*_f_	1	0.84	0.73	0.92
*Q*_10-vmax_	1	1.86	1.86	1.86
*Q*_10-Km_	1	1.40	1.40	1.40
*Q*_10[Soils]_	1	0.94	1.10	0.99
[*Soils*]_20_	μm	12	24	12
*K*_m20_	μm	57	70	60
[*Soils*]_20_/*K*_m20_	1	0.21	0.34	0.20
*CUE*_20_	1	0.20	0.50	0.20
*CUE*_30_	1	0.17	0.36	0.18
*Q*_10-R%_	1	1.04	1.27	1.02

Sensitivity analysis was employed to find the variables that exerted the largest influence on *Q*_10i_. We then investigated the effects of changing [*S*]_20_/*K*_m20_ on *Q*_10i_, with and without considering *Q*_10-R%_ by setting it to 1 or the estimated value. Finally, the evolution of *Q*_10t_ for cumulative SOM mineralization was simulated at a 1 h time step over 720 h of incubation according to Equation (13), with the assumption that *D_f_* (which indicates the effects of warming on microbes) decreases linearly with time over 240 h, due to the gradual reduction of microbial biomass or enzyme pools.

## Results

### Mineralization of soil organic matter pools and their *Q*_10_

**Figure 2:**
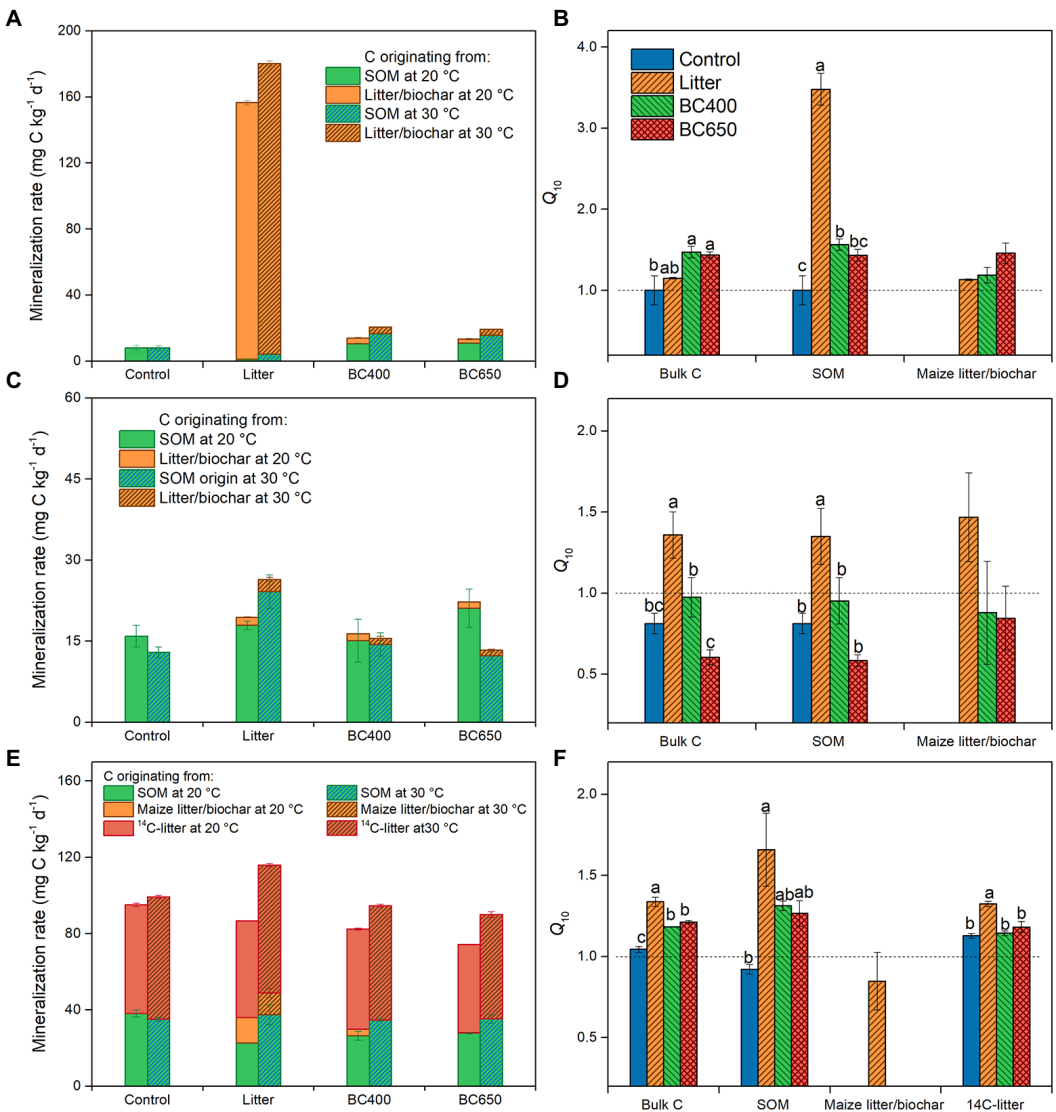
Mineralization of organic C pools in soil **(A–C)** and their *Q*_10_ **(D–F)** at the early incubation stage **(A,D)**, and at the late incubation stage without **(B,E)** and with **(C,F)** secondary litter amendment. Error bars represent standard errors (*n* = 3). Lowercase letters above bars in **(D–F)** indicate significant differences between the treatments (*p* < 0.05). There were initially four treatments (Control, Litter, BC400, and BC650). At the late incubation stage, part of the soils kept their earlier treatments, whereas the remaining all received secondary fresh litter (^14^C-labeled) in addition to their prior amendments. Both the early- and late-stage soils were warmed over short periods by incubating soils at 20°C (unwarmed) and 30°C (warmed).

At the initial stage of soil incubation, decomposition of maize litter dominated CO_2_ efflux under litter addition, where SOM mineralization was even lower than that in the unamended control soil (Figure 2A). In contrast, biochar amendments accelerated SOM mineralization by 30% relative to that of the control at 20°C. Raising the incubation temperature to 30°C resulted in a much higher *Q*_10_ (3.5) of SOM in the litter-amended soils than in the control (*Q*_10_ close to 1) and biochar-amended soils (*Q*_10_ = 1.5, Figure 2B).

After 417 days of incubation, litter decomposition greatly declined, as 58% of amended maize litter was already decomposed (data not shown), and SOM decomposition contributed 92% to total soil CO_2_ emission (Figure 2C). SOM mineralization in the litter-amended soils became higher than that in the control, particularly at 30°C. The *Q*_10_ of total C and SOM mineralization was higher under litter amendment (*Q*_10_ = 1.4 for total C and SOM) than in the control or biochar-amended soils (Figure 2D). The mass-specific respiration (*R*_mass_, for total CO_2_ emission) was similar between soils with different amendments at 20°C, but was significantly higher in litter-amended soils at 30°C (Table 2). In addition, the temperature response of *R*_mass_, expressed as the ratio of *R*_mass_ at 20°C to 30°C (*R*_30/20_), was significantly larger under the litter amendment.

The secondary addition of ^14^C-labeled rice litter on day 417 greatly increased total C and SOM mineralization, as well as *R*_mass_, in all soils, with or without prior amendments (Table 2; Figure 2E). For soils that received fresh litter, *R*_mass_ was highest in soils with prior maize litter addition at 20°C and 30°C (Table 2). *Q*_10_ of SOM was increased by the secondary litter addition from 0.6–1.3 to 0.9–1.6, with the highest *Q*_10_ (1.6) in the original maize-litter-amended soils (Figure 2F). The *Q*_10_ for the newly added rice litter *per se* was also higher in soils with prior maize litter addition (1.3) than that in the original control or biochar-amended soils (1.1). The temperature response of *R*_mass_ was higher in soils with secondary litter addition (1.3) than in soils without the secondary addition (2.2).

**Figure 3:**
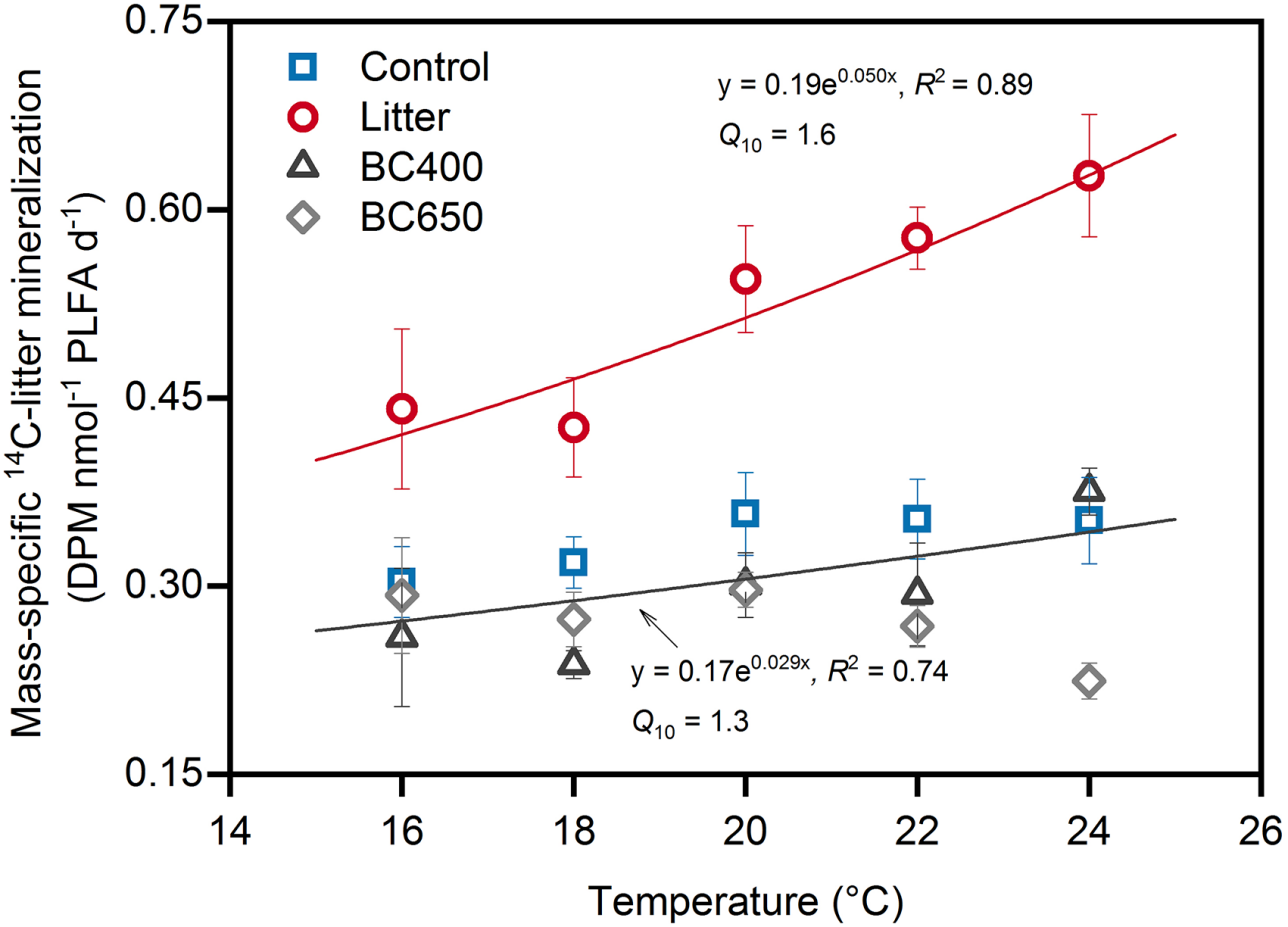
Decomposition of the secondarily added ^14^C-labeled rice litter at sequentially changing incubation temperatures. The decomposition was expressed as microbial-mass-specific rate. Error bars identify standard errors (*n* = 3). The sequential-warming experiment was conducted for soils (with original treatments of Control, Litter, BC400 or BC650) that had been incubated at 20°C for 417 days, after which fresh ^14^C-labeled rice litter was added before incubation at 20°C for another 48 days. The two fitted exponential equations describe the relationship between decomposition rate and temperature. The decomposition rates of ^14^C-litter were similar between the control and soils with prior amendments of BC400 and BC650; thus only one equation was given for these treatments.

The results of the sequentially increasing temperature incubation revealed an exponential relationship between the newly added ^14^C-labeled rice litter decomposition (microbial-biomass-specific) and temperature in the original maize-litter-amended soil (Figure 3), resulting in a relatively high *Q*_10_ of 1.6. In comparison, mass-specific ^14^C-litter decomposition rates and the corresponding *Q*_10_ (1.3) were lower in the original control and biochar-amended soils, particularly within the temperature range of 20–24°C. The ^14^C-litter decomposition was similar in the prior control and biochar-amended soils.

### Temperature dependence of enzyme kinetics and activities

All enzyme activities, and their kinetic parameters *V*_max_ (Supplementary Figure S1) and *K*_m_ (Supplementary Figure S2; except for LAP at the late incubation stage), showed exponential relationships with assay temperature (8–32°C). *Q*_10_ for *V*_max_ had a mean value of 1.8, which was larger than that for *K*_m_ (1.4). At the early and late incubation stages without secondary litter addition, *V*_max_ was generally highest under maize-litter addition (*p* < 0.05). Overall, *K*_m_ was not affected by these amendments. The *Q*_10_ of *V*_max_ and *K*_m_ of all three enzymes across assay temperatures were similar between amendments at either the early or late incubation stage, with or without secondary litter addition.

**Figure 4:**
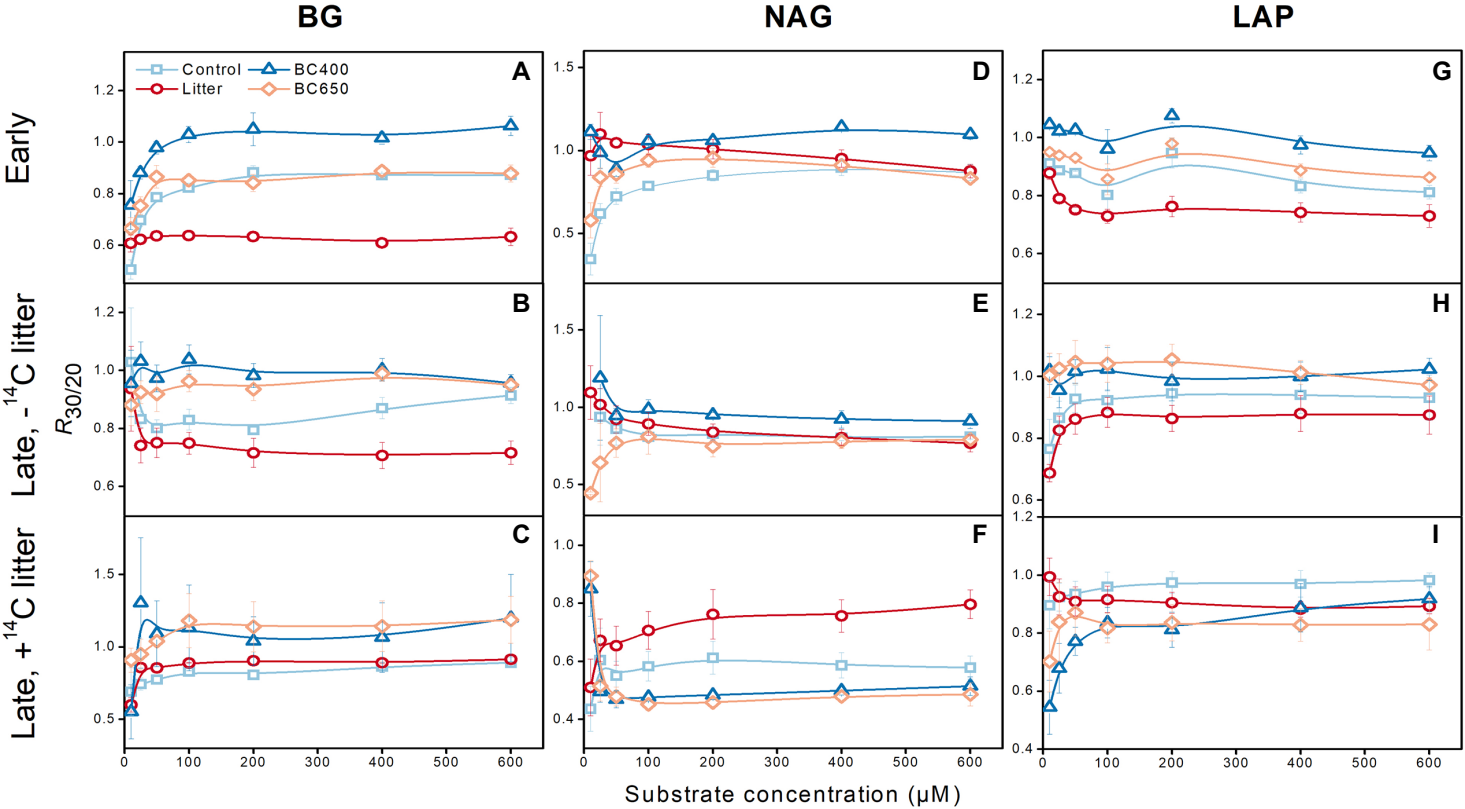
Effects of warming on enzyme activities for β-glucosidase (BG, **A–C**), N-acetyl-β-glucosaminidase (NAG, **D–F**) and leucine aminopeptidase (LAP, **G–I**). The warming effect was expressed as the ratio of enzyme activities (averaged over assay temperatures) in warmed (incubated at 30°C) versus unwarmed soils (incubated at 20°C; *R*_30/20_). Error bars represent standard errors (*n* = 3). At the early incubation stage there were four treatments (Control, Litter, BC400, and BC650). At the late incubation stage, part soils kept their earlier treatments, whereas the remaining received secondary fresh litter (^14^C-labeled), in addition to their prior treatments. Both the early-and late-stage soils were subject to warming.

In most cases, warming from 20 to 30°C decreased *V*_max_ (data not shown) and enzyme activities (Figure 4). The magnitude of the decreases in enzyme activities (expressed as *R*_30/20_, the ratio of activities at 30 to 20°C) was greater in the maize-litter-amended soils than in biochar-amended soils. *R*_30/20_ ranged between 0.5 and1. Notably, *R*_30/20_ was mostly close to 1 under the two biochar amendments (BC400 in particular), i.e., declines in enzyme activities were minimal, but *R*_30/20_ could be as low as 0.6–0.7 in maize-litter-amended soils. Overall, secondary litter addition decreased *R*_30/20_ (particularly for NAG, with *R*_30/20_ decreasing to approximately 0.5) for soil with or without prior amendments. For all the enzymes, *K*_m_ showed no consistent response to warming (Supplementary Figure S2). The *Q*_10_ of *V*_max_ and *K*_m_ (Supplementary Figures S1, S2) were similar between the warmed and unwarmed soils (both *p* > 0.05).

### PLFA composition and temperature stress indicators

**Figure 5:**
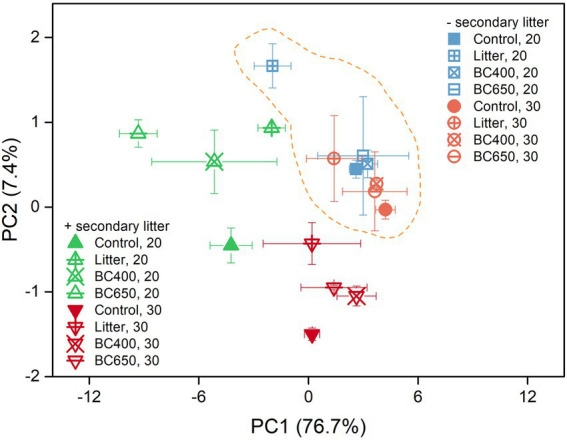
Ordination graph from the principal component analysis (PCA) of soil phospholipid fatty acid (PLFA) profiles at the late incubation stage, with or without secondary litter amendment. The percentage of variation explained by each principal component is given in the brackets beside each axis. Error bars indicate standard errors (*n* = 3). Soil under all four treatments (Control, Litter, BC400, and BC650) were secondarily amended with fresh rice litter or kept their original treatments at the late incubation stage. All soils were then placed at 20°C (unwarmed) and 30°C (warmed) to mimic a short-term warming event. Samples surrounded by the dashed line did not receive secondary litter addition.

**Figure 6:**
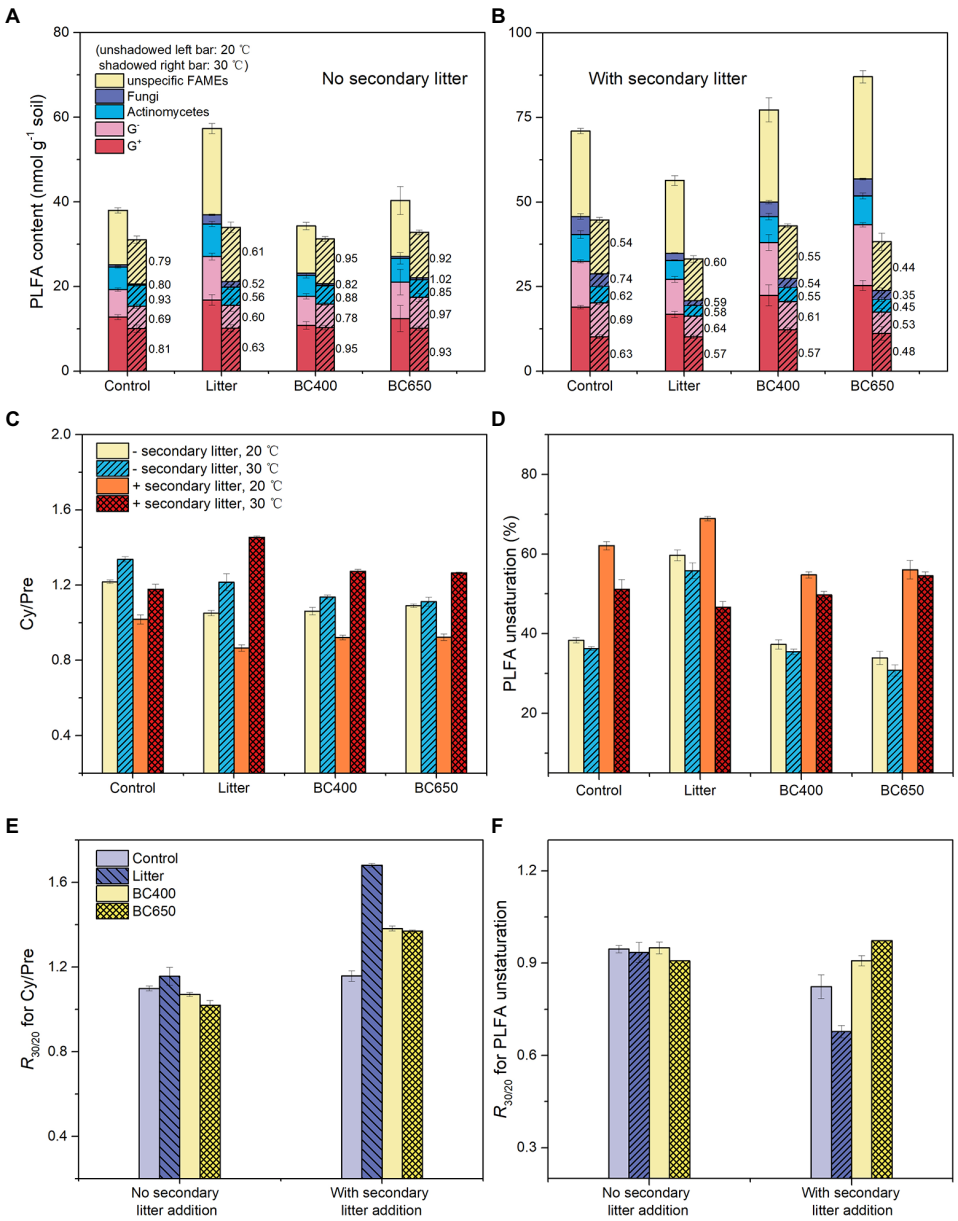
Phospholipid fatty acid (PLFA) content and temperature stress indicators calculated from PLFA data at the lateincubation stage: **(A,B)** PLFAs belonging to various microbial groups, with their sensitivities to warming (the ratio of levels at 30°C–20°C) given by the number alongside the bars; **(C,D)** ratio of cyclopropyl fatty acids (cy17:0 and cy19:0) to their precursors (Cy/Pre) and PLFA unsaturation; **(E,F)** sensitivities of Cy/Pre and PLFA unsaturation to warming (ratio of levels at 30°C–20°C, *R*_30/20_). Soil under four prior treatments (Control, Litter, BC400, and BC650) with or without secondary addition of fresh rice litter were placed at 20°C (unwarmed) and 30°C (warmed) to mimic a short-term warming event. Error bars indicate standard errors (*n* = 3). Note that the contents of all PLFAs were lower at 30°C than 20°C.

The PLFA data indicated substantial differences in the microbial composition between the soils with maize litter and biochar. At the initial incubation stage, maize litter addition caused a 300% increase in total PLFA and a 13-fold increase in fungal PLFAs (Supplementary Table S2). In contrast, the total PLFA and PLFA composition were similar between the control and soils amended with BC400 or BC650. After 417 days, the PCA results demonstrated remarkable differentiation in microbial composition in response to warming and secondary litter addition (Figure 5). Raising the incubation temperature reduced the total PLFA content (Table 2) and PLFA markers of nearly all microbial groups (Figures 6A,B), regardless of the presence of secondary litter input. Without secondary litter addition, the magnitudes of such reductions, reflected in the ratio of PLFA at 30–20°C (*R*_30/20_), were largest in soils receiving maize litter. Amending with fresh litter lowered *R*_30/20_ in all soils, indicating that the total PLFA became more sensitive to warming.

Two calculated PLFA indicators of temperature stress, the Cy/Pre ratio and PLFA unsaturation, responded significantly to warming and secondary litter addition (Figures 6C,D). Cy/Pre increased with warming, with the magnitude of increase (the ratio at 30–20°C) being larger following secondary litter addition (Figure 6E). The PLFA unsaturation was greatly increased by secondary litter addition, but dropped after warming (Figure 6F).

### Modeling analysis of factors influencing *Q*_10_ of SOM

**Figure 7:**
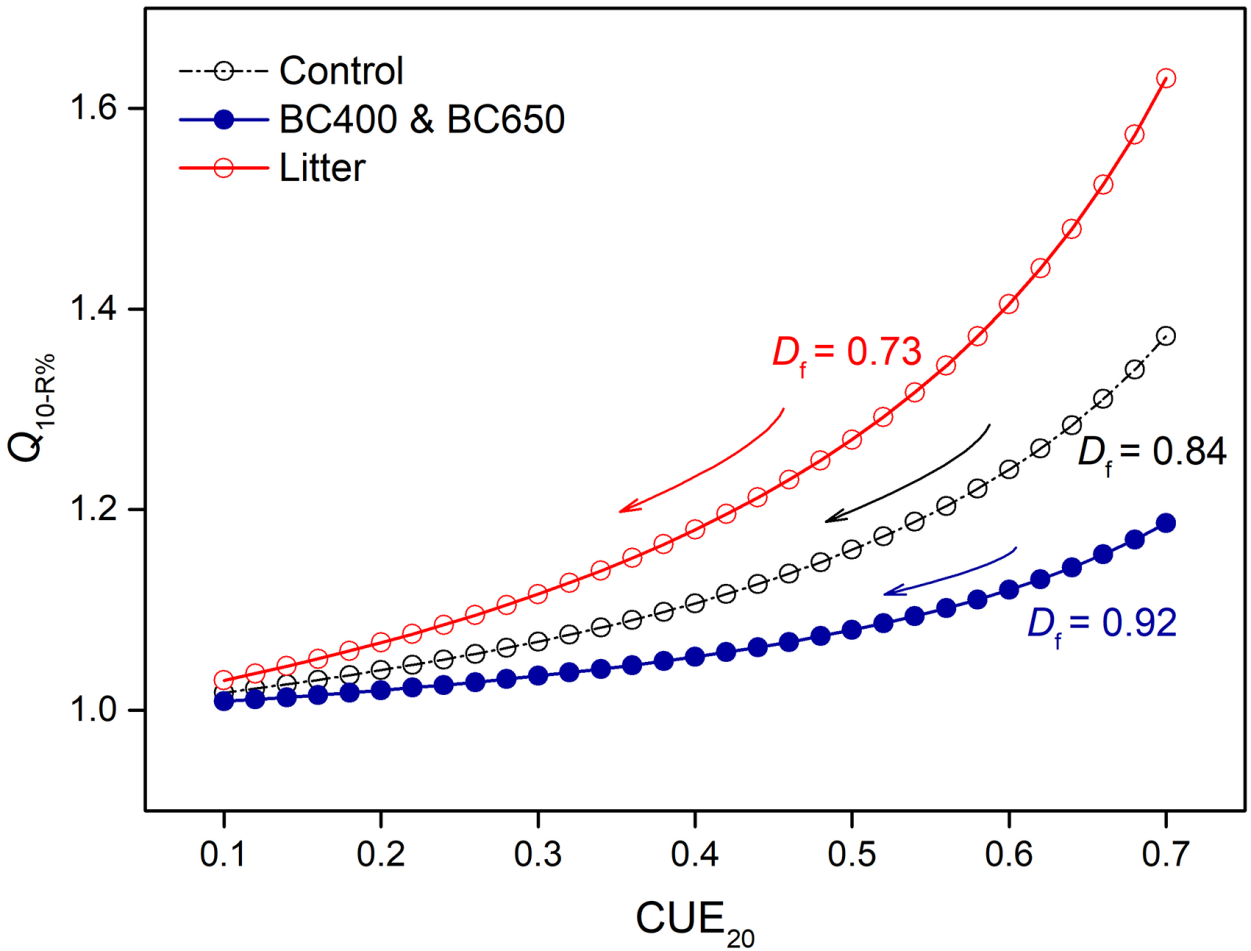
Simulated *Q*_10-R%_ as a function of microbial carbon use efficiency at 20°C (CUE_20_), depending on soil amendments (litter or litter-made biochar). *Q*_10-R%_ reflects the loss of assimilated C due to decreasing CUE under warming, and was calculated according to Equation (14). *D*_f_ indicates the magnitude of CUE declines as temperature increases from 20 to 30°C. Lower *D*_f_ is used for litter-amended soils, where microbial biomass and enzyme pools decrease more under warming. Note that *Q*_10-R%_ was consistently higher in soils with lower *D*_f_, and at a fixed *D*_f_ the loss of assimilated C becomes less temperature-sensitive (i.e., *Q*_10-R%_ decreases) as CUE decreases.

The default parameter values used in the modeling analysis are listed in Table 3. Most of these variables (*D_f_*, *Q*_10-vmax_, *Q*_10-Km_, and *Q*_10[S]_) were well constrained by our enzyme data (Supplementary Figures S1, S2), and DOC in unwarmed and warmed soils (Table 2). The substrate availability ([*S*]_20_) in the control soil was approximated by fitting BG activity to Equation 16. The [*S*]_20_ of the litter-amended soil was assumed to be double that of the control, to simulate the priming of litter on SOM depolymerization, although the measured DOC (mainly derived from SOM) was only 50% higher under litter addition. *Q*_10-R%_, which measured the increasing proportion of microbial assimilated C loss *via* cell respiration with warming, was a function of *D_f_* and CUE at 20°C (Equation 14). However, the CUE was not measured and had to be estimated. Reported CUE values for litter could be as high as >0.6 (Lashermes et al., 2016; Joergensen and Wichern, 2018; Sauvadet et al., 2018), but we adopted a value of 0.5 for the litter-amended soils, because CUE might have declined with time. For the control and biochar amendments, we used a much lower CUE value (0.2), as reported by Spohn et al. (2016) for a C-poor subsoil, which resembled C-depleted soils after 417 days of incubation. It is reasonable to use a higher CUE for microbes living on energy-rich litter than for those using only SOM (Joergensen and Wichern, 2018). This resulted in a higher *Q*_10-R%_ under litter amendment compared to those under the control and biochar amendments. This between-amendment pattern of *Q*_10-R%_ was mainly dictated by the lower *D_f_* for litter amendments, and was robust to the estimated CUE values for specific amendments across a wide range (Figure 7).

**Figure 8:**
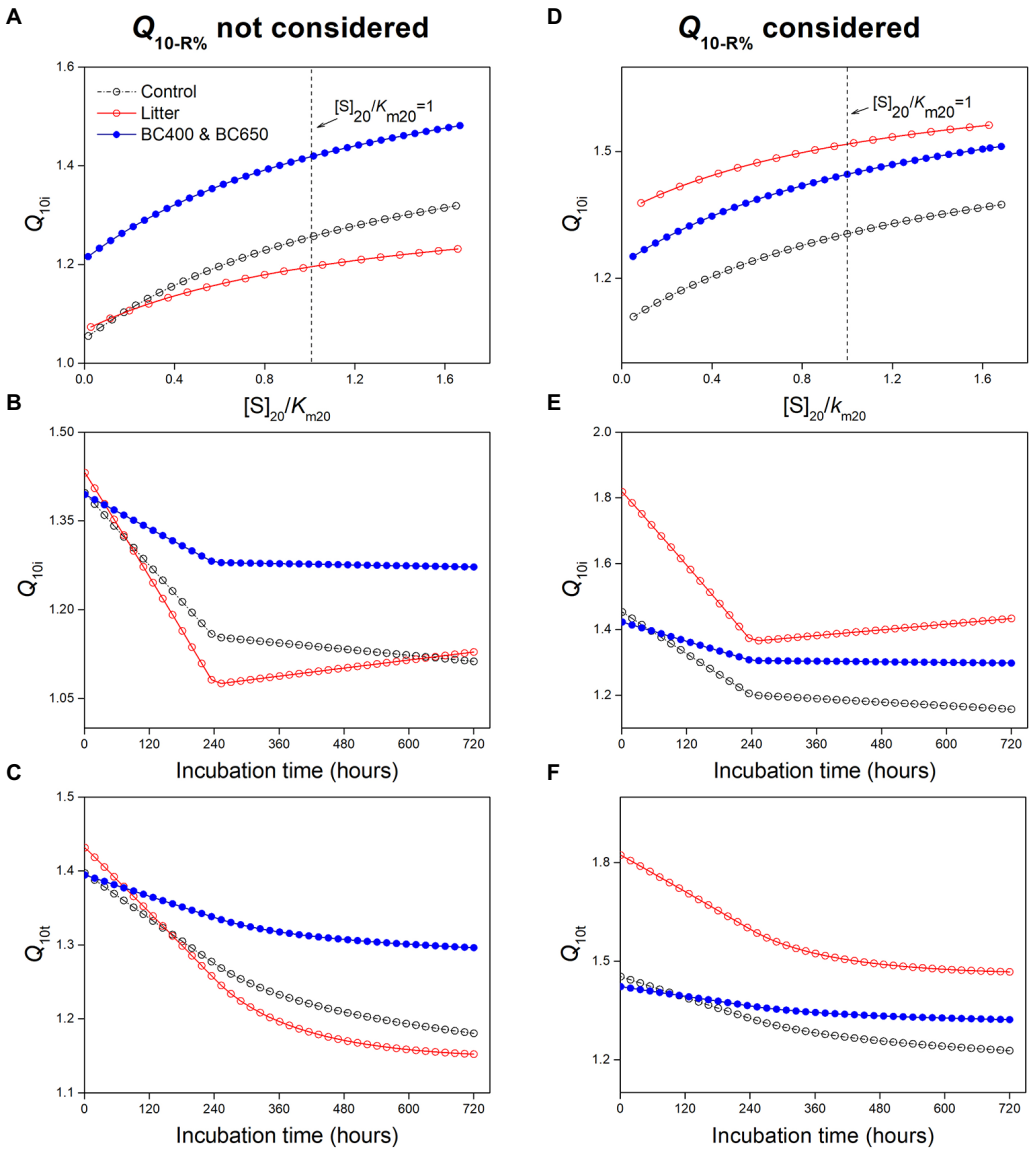
Results of the modeling analysis: **(A–C)** effects of [*S*]_20_/*K*_m20_ (ratio of SOM-derived substrate concentration to *K*_m_ at 20°C) on instantaneous temperature sensitivity (*Q*_10i_) of SOM **(A)**, the modeled *Q*_10i_ of SOM using parameter values in Table 3 **(B)**, and cumulative temperature sensitivity (*Q*_10t_) of SOM mineralization with time based on modeled *Q*_10i_ **(C)** without considering *Q*_10-R%_ in Equation (12); **(D–F)** relationship between *Q*_10i_ and [*S*]_20_/*K*_m20_ **(D)**, and the modeled *Q*_10i_ **(E)** and *Q*_10t_ **(F)** of SOM mineralization with *Q*_10-R%_ set to values in Table 3.

We mainly focused on the influence of substrate availability (indicated by [*S*]_20_/*K*_m20_) and microbial physiological characteristics (mainly the temperature-dependence of CUE, which determined *Q*_10-R%_) on the temperature sensitivity of SOM decomposition. First, we investigated the effects of substrate availability by varying [*S*]_20_/*K*_m20_ without considering *Q*_10-R%_ (with *Q*_10-R%_ set as 1), and found that the instantaneous *Q*_10i_ for SOM increased with [*S*]_20_/*K*_m20_ (Figure 8A). However, *Q*_10i_ was insensitive to [*S*]_20_/*K*_m20_ (Table 4). Even if we assumed a two-fold substrate content under litter amendment relative to that of the control (Table 3), this only slightly affected *Q*_10i_ (1.11 and 1.13 for control and litter-amended soils, respectively). The corresponding simulated temperature sensitivities for SOM (instantaneous and cumulative) were lowest in litter-amended soils (Figures 8B,C), which was contradictory to our experimental observation. Therefore, the higher substrate availability in litter-amended soils alone could not account for the greater temperature sensitivity of SOM mineralization.

**Table 4:** Sensitivity analysis of *Q*_10i_ values (instantaneous temperature sensitivity, Equation 12) to key parameters depending on biochar and maize litter addition.

Parameter	Input range		Sensitivity^a^		
	Lower	Upper	Control	Litter	BC400 & BC650
*D*_f_	0.4	1	1.00	1.00	1.00
*Q*_10_vmax_	0.5	5	1.00	1.00	1.00
*Q*_10_Km_	0.5	5	0.87	0.79	0.87
*Q*_10[S]_	0.5	2	0.86	0.80	0.87
[*S*]_20_/*K*_m20_	0.01	2	0.05	0.03	0.04
*Q*_10-R%_	1	3	1.00	1.00	1.00

The input ranges of these parameters cover all possible values. Where lower and upper input refer to the lower and upper bounds of the input values of each parameter, and lower and upper outputs are the corresponding *Q*_10i_, respectively.

a Sensitivity of *Q*_10i_ to an input parameter was assessed by: $\mathrm{Sensitivity}=\frac{\log_{10}\left(\mathrm{upper output}\right)-\log_{10}\left(\mathrm{lower output}\right)}{\log_{10}\left(\mathrm{upper input}\right)-\log_{10}\left(\mathrm{lower input}\right)}$ following Allison et al. (2010).

However, when we considered a higher *Q*_10-R%_ (i.e., larger warming-induced declines in microbial CUE) in litter-amended soils, the temperature sensitivities of SOM exceeded those in the control and biochar-amended soils (Figures 8D–F). The resultant pattern of *Q*_10t_ for SOM across amendments (i.e., Control ~ BC400 ~ BC650 < Litter) agreed with our experimental observations (Figure 2D). This could be because that *Q*_10i_ of SOM was much more sensitive to *Q*_10-R%_ than to [*S*]_20_/*K*_m20_ (sensitivity: 1 versus 0.04 for *Q*_10-R%_ and [*S*]_20_/*K*_m20_, respectively; Table 4). Based on these results, *Q*_10-R%_ was a more important determinant of the temperature sensitivity of SOM decomposition than substrate availability ([*S*]_20_/*K*_m20_).

## Discussion

### Less temperature-tolerant microbial communities In soil with litter amendments

Soil enzymes may adapt to warmer environments with rigid structures to enable better substrate affinity (Bradford et al., 2008; German et al., 2012), which tends to increase the *Q*_10_ of *V*_max_ but decreases that of *K*_m_, as hypothesized by Allison et al. (2018). The *Q*_10_ for *V*_max_ and *K*_m_, however, remained stable under short-term warming (Supplementary Figures S1, S2), suggesting little thermal adaptation of the enzyme structure and function. It is plausible that the new sets of isoenzymes produced in warmed soils maintain a relatively constant *Q*_10_ (Razavi et al., 2016). This may also account for the insignificant warming effects on *K*_m_ (Supplementary Figure S2).

Nevertheless, warming decreased *V*_max_ and enzyme activities (Figure 4), which could be associated with reductions in microbial biomass (Figures 6A,B) rather than enzyme thermal adaptation. *V*_max_ in soil usually reflects the enzyme pool size (Wallenstein et al., 2010), which, in turn, is linked to microbial biomass (Allison et al., 2010). Indeed, for soils at the late incubation stage, warming decreased the total PLFA content by approximately 24%, at a magnitude comparable to that for enzyme activities (15%) and *V*_max_ (18%). There were also positive correlations between warming-induced declines in PLFA and enzyme activities, particularly for NAG and LAP (Supplementary Figure S3). Notably, warming reduced microbial biomass more in the soil with litter (Figures 6A,B; Table 2), where microorganisms grew actively. These results suggest that microbial communities activated by litter were less tolerant to high temperatures than inactive microbes under biochar amendments.

Warming-induced enzyme denaturation should not be the major mechanism of *V*_max_ decline, because the loss of extracellular enzyme activities occurs slowly in soil owing to the stabilization of enzyme molecules on mineral surfaces (Allison, 2006; Schimel et al., 2017). For instance, it is very unlikely that warming-induced denaturation decreased the activities of enzymes such as NAG by nearly 50% following secondary litter input (Figure 4F). In addition, denaturation reduces the binding sites of enzymes, weakens substrate-enzyme affinity, and decreases *K*_m_. However, we detected a relatively similar *K*_m_ between warmed and unwarmed soils, with or without litter addition. Decreased microbial biomass and enzyme production together with accelerated enzyme turnover (Conant et al., 2011) are more important controlling factors than denaturation for the declining *V*_max_ and apparent enzyme activities with soil warming.

Decreased microbial CUE or growth efficiency at higher temperatures, due to higher maintenance energy costs or waste metabolism (Bradford, 2013), accounts for the warming-induced reduction in microbial biomass (Tucker et al., 2013). This was supported by the overall higher mass-specific respiration at 30°C than at 20°C at the late incubation stage (Table 2). Moreover, warming increased the microbial synthesis of cyclopropyl and saturated fatty acids (Figures 6C,D), which can counter membrane fluidity at high temperatures (Suutari and Laakso, 1994; Wixon and Balser, 2013), but is energetically expensive (Zogg et al., 1997). Because of the trade-off between microbial growth and stress tolerance (Malik et al., 2020), this unavoidably reduces resource allocation to microbial growth, thereby decreasing microbial growth and CUE. Higher temperatures may also increase microbial death rates (Joergensen et al., 1990).

The mechanisms underlying the lower heat tolerance of actively growing microbial communities under litter input are still not clear. We hypothesize that the growing r-strategists stimulated by labile litter (Loeppmann et al., 2016) are less stress-tolerant than inactive and starving microbes (oligotrophs or K-strategists; Lipson, 2015) under biochar amendments. Starvation makes microbial species (mainly K-strategists) more resistant to stress (e.g., heat, UV; Nyström et al., 1992; Hartke et al., 1998). Specific proteins and lipids induced by starvation are produced to cope with environmental stress (Hartke et al., 1998). On the other hand, the r-strategists under C-abundant conditions may mainly invest energy into growth rather than stress resistance. Indeed, microorganisms growing on litter demonstrated lower cyclopropyl and saturated fatty acid levels (mainly at 20°C, Figures 6C,D), suggesting that they synthesized fewer heat-resistant compounds than K-strategists (Wixon and Balser, 2013). Overall, the distinct microbial life strategies, and corresponding energy allocation tradeoff between growth and stress resistance, might underlie microbial heat tolerance under litter and biochar amendments.

### Increased resistance of enzymes and microbes to warming under biochar amendments

Biochar addition preserved enzyme activities at high temperatures, as evidenced by the lower warming-induced decline in enzyme activities in biochar-amended soils relative to the control or litter-amended soils (Figure 4). Biochar stimulated the biosynthesis of saturated fatty acids, which is beneficial to microbial temperature resistance (Wixon and Balser, 2013), as seen from the lower PLFA unsaturation in unwarmed biochar-amended soils receiving secondary litter addition (Figure 6D).

In addition, biochar may create biologically favorable soil space (microbial niches) near its surfaces, i.e., the “charosphere” formed by the adsorption of water, nutrients and biomolecules (Luo et al., 2013; Quilliam et al., 2013). The charosphere may have contributed to the persistence of enzyme activity in the biochar-amended soils under warming conditions.

### Lower *Q*_10_ of soil organic matter decomposition under biochar than under litter

*Q*_10_ of SOM decomposition is often increased by the addition of labile substrate (Gershenson et al., 2009; Liu et al., 2021). However, the effects of labile C addition on the temperature sensitivity of SOM versus FOM remain poorly understood (Wei et al., 2021). Herein, *Q*_10_ of both SOM and FOM increased with litter input, which was not observed with biochar addition (Figures 2, 3). Therefore, using litter-made biochar as a soil amendment instead of litter may lower the responses of both SOM and FOM decomposition to warming.

Substrate availability (i.e., item [*S*] in the Michaelis–Menten equation) increases the *Q*_10_ of soil CO_2_ emission, which has frequently been emphasized previously (Pang et al., 2015; Liu et al., 2021). The generally low [*S*] in soils is a major constraint on the temperature sensitivity of many C cycling processes (Davidson et al., 2006). However, only a few studies have focused on the relationship between substrate availability and *Q*_10_ of SOM under fresh C inputs (Wei et al., 2021). Zhu and Cheng (2011) ascribed the increased *Q*_10_ of SOM with plant rhizodeposits to increased SOM-derived substrate availability, as stimulated by enzyme production. Despite more SOM-originating substrates (DOC, Table 2) in the litter-amended soils, we consider this to be a minor contributor to the *Q*_10_ of SOM. This is because *Q*_10_ is directly related to the substrate-*K*_m_ ratio ([*S*]/*K*_m_) rather than the substrate content *per se* ([*S*]; Equation 12). The *Q*_10_, however, was insensitive to [*S*]/*K*_m_ values in our modeling (Table 4; Figure 8A). Furthermore, [*S*]/*K*_m_ for SOM generally had small values (approximately 0.2 here), as SOM-derived [*S*] was commonly low relative to *K*_m_ (Larionova et al., 2007; Allison et al., 2010), and was unlikely to be substantially improved given the low energy availability of SOM (Gunina and Kuzyakov, 2022).

Our modeling suggested that *Q*_10_ of SOM was much more sensitive to warming-induced declines in microbial CUE (and hence loss of assimilated C with temperature, *Q*_10-R%_) than to substrate availability (Figure 8). This is partly because in our modeling (Equation 12), *Q*_10_ had a linear relationship with *Q*_10-R%_, but a saturating (Michaelis–Menten-like) relationship with substrate availability ([*S*]/*K*_m_). In addition, if only substrate availability was considered, the modeled *Q*_10_ for SOM would be lowest under the litter amendment (Figures 8A–C), which was opposite to our experimental observations (Figure 2).This was because microbial biomass and enzyme activities declined the most with warming in litter-amended soils, which had strong negative impacts on *Q*_10_ that could not be counteracted by the slightly higher [*S*]/*K*_m_ than under biochar amendments. The litter-amended soils had the highest simulated *Q*_10_ only when assigned the highest *Q*_10-R%_ (Figure 8F). This was reasonable because their mass-specific respiration and microbial biomass was much more sensitive to temperature elevation (Table 2; Figure 6), supporting that warming decreased microbial C utilization for growth (i.e., CUE; Li et al., 2019) to greater extents in litter-amended soils. However, the importance of microbial physiology to *Q*_10_ of SOM decomposition has often been neglected in previous studies (Zhu and Cheng, 2011; Su et al., 2022). Recently, Xu et al. (2022) found that vegetable field soils in warmer regions tended to have lower CUE and higher *Q*_10_ of SOM decomposition, highlighting the possible control over *Q*_10_ by CUE. Therefore, *Q*_10_ may be modeled as a function of microbial physiological parameters such as CUE.

The regulation of temperature sensitivity by microbial CUE may also account for the high *Q*_10_ of FOM inputs in soils with earlier litter additions. Following secondary litter addition, substrate availability was similar between all soils (DOC, Table 2). However, the *Q*_10_ of mass-specific decomposition of FOM was evidently higher in soils with earlier maize-litter amendments (Figure 3), suggesting that CUE in litter-amended soils might decline more with warming than in biochar-amended soils (Liu et al., 2020). Presumably, microbial community composition in prior litter-amended soils was more dominated by the temperature-intolerant r-strategists, making microbial CUE more temperature-sensitive even after FOM inputs. On the other hand, CUE of biochar-amended soils might be higher under warming as it could be facilitated by the beneficial effects of the “charosphere” on microbial cells and enzyme molecules (Luo et al., 2013; Quilliam et al., 2013).

**Figure 9:**
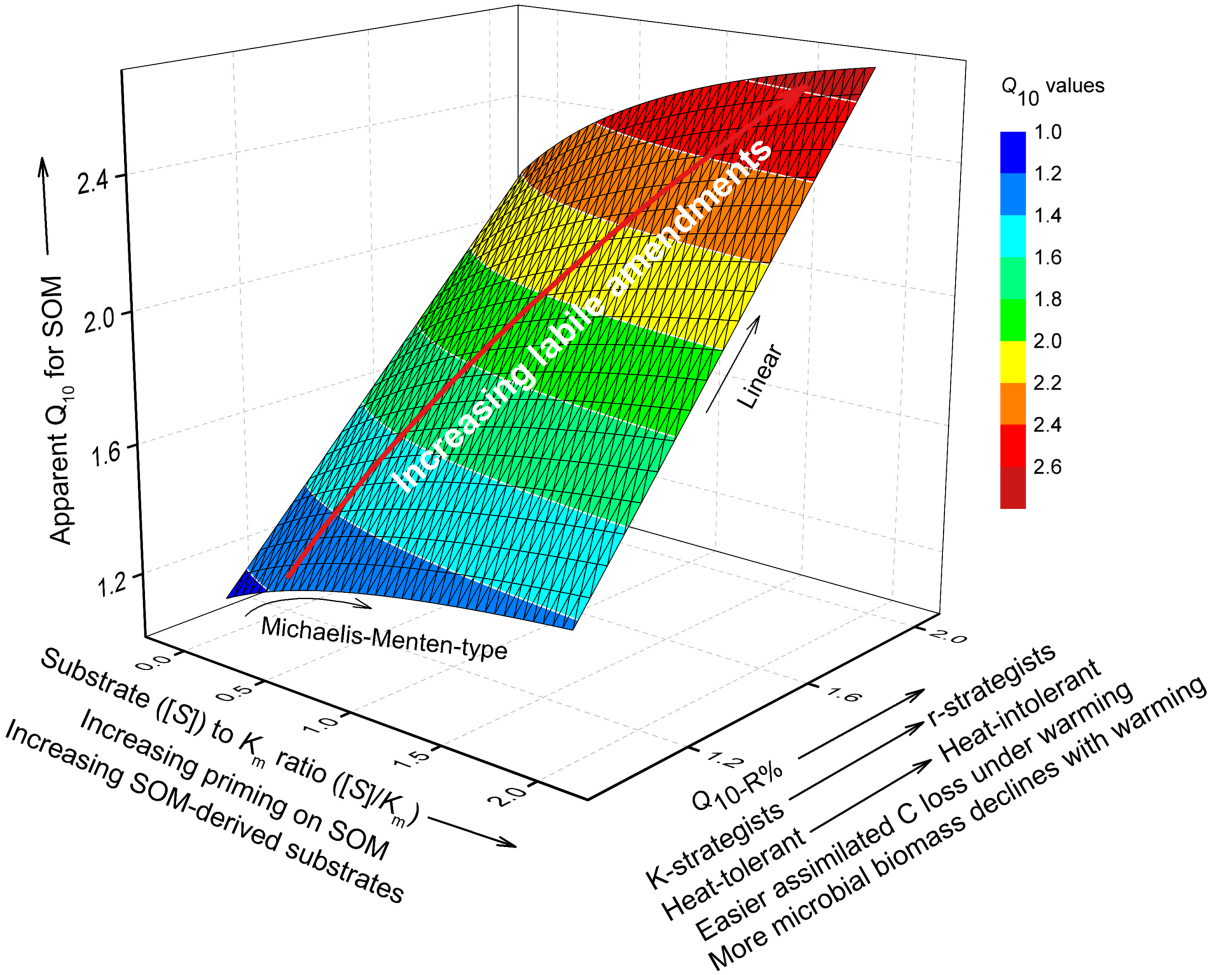
Conceptual figure showing the contribution of substrate availability and microbial physiological parameters on apparent *Q*_10_ of SOM decomposition, with the priming effect considered. The influence of substrate availability ([*S*]) is manifested in the item [*S*]/*K*_m_ (Equation 12), i.e., the ratio of substrate availability to half saturation constant (*K*_m_) in Michaelis–Menten kinetics. [*S*]/*K*_m_ may increase due to the priming of SOM depolymerization by labile C inputs. The influence of microbial physiology on *Q*_10_ is mainly through *Q*_10-R%_, i.e., warming-accelerated loss of assimilated C from microbial cells. *Q*_10-R%_ increases with the proliferation of r-strategists under labile C inputs, which leads to higher microbial biomass that is less resistant to warming, and hence more easily loses assimilated C at high temperatures. According to the 3D shape, the *Q*_10_ of SOM is much more sensitive to increasing *Q*_10-R%_ than to increasing [*S*]/*K*_m_. The white lines are the contours of *Q*_10_. The red arrow indicates the trajectory of *Q*_10_ changes with [*S*]/*K*_m_ and *Q*_10-R%_ owing to increasing labile amendments to soil.

Overall, through modeling analysis, we tentatively postulate that under exogenous C inputs, microbial physiology may outweigh substrate availability in controlling *Q*_10_ of the SOM (Figure 9). Added organic C should have a limited influence on substrate release from SOM, a mixture of substances with low energy availability that is consequently unfavorable to microbes (Gunina and Kuzyakov, 2022). In comparison, substantial changes in microbial life strategies (e.g., r versus K), as well as their heat tolerance, may occur under labile C inputs, greatly modifying the final *Q*_10_ for SOM decomposition. Future modeling of the temperature sensitivity of SOM decomposition should place greater emphasis on the microbial physiological changes (particularly decreasing CUE) in response to both warming and substrate availability.

## Conclusion

Compared with biochar amendment, litter increased the rates and *Q*_10_ of both SOM and FOM decomposition. Litter addition stimulated microbial growth and activities to yield more extracellular enzymes, but the actively growing microbes were less resistant to warming than inactive microbes. Biochar had almost no effect on microbial growth, but made enzyme activities more resistant to high temperatures. This was possibly linked to the existence of the “charosphere,” i.e., the biologically favorable space in the vicinity of biochar surfaces, due to the adsorption of water, nutrients, enzymes and substrates. Theoretically, greater warming-induced losses of microbial biomass and enzyme pools in litter-amended soils should lower *Q*_10_. However, the litter-amended soils still had higher *Q*_10_ of SOM and FOM than biochar-amended soils, because warming accelerated microbial C loss (as reflected in the mass-specific respiration) to greater extents under litter inputs due to dominance of temperature-intolerant r-strategists. The acceleration of assimilated C loss also explains why litter-amended soils showed greater magnitudes of microbial biomass decline in response to warming. Despite the increased SOM-derived substrate availability by priming under litter inputs, our modeling results suggest this as a lesser contributor to the higher *Q*_10_ of litter-amended soils than the changing microbial physiology under warming (i.e., microbes more easily lose C by respiration at higher temperatures). Overall, we highlighted soil microbial physiological characteristics (e.g., microbial biomass, enzyme pools, mass-specific respiration, CUE, and their temperature dependence) as critical determinants of the temperature sensitivity of SOM decomposition, in addition to previously emphasized substrate availability. Whether the greater microbial vulnerability to warming under labile litter inputs is associated with the stress intolerance of r-strategists merits further investigation. Overall, we propose that rather than returning pure straw to the soil (a common agricultural practice), incorporation of straw-made biochar, or a combined application of biochar and straw, may be a better option to slow down SOM decomposition in agroecosystems in a warming climate.

## Data availability statement

The raw data supporting the conclusions of this article will be made available by the authors, without undue reservation.

## Author contributions

JC, CF, and ZD conceived and designed the research. JC, TG, and MN conducted the experiments and soil analysis. JC wrote the manuscript. JC, YK, and SA analyzed the data and edited the manuscript. All authors contributed to the article and approved the submitted version.

## Funding

This research was funded by the National Natural Science Foundation (grant number: 31872695), and supported by the RUDN University Strategic Academic Leadership Program and the High-level Personnel Office of Nantong University (135421609072).

## Conflict of interest

The authors declare that the research was conducted in the absence of any commercial or financial relationships that could be construed as a potential conflict of interest.

## Publisher’s note

All claims expressed in this article are solely those of the authors and do not necessarily represent those of their affiliated organizations, or those of the publisher, the editors and the reviewers. Any product that may be evaluated in this article, or claim that may be made by its manufacturer, is not guaranteed or endorsed by the publisher.

## Supplementary material

SUPPLEMENTARY FIGURE S1 Relationship between log(*V*_max_) and assay temperature of enzyme kinetics in unwarmed (at 20°C) and warmed soils (30°C). Error bars indicate standard errors (*n* = 3).

SUPPLEMENTARY FIGURE S2 Relationship between log(*K*_m_) and assay temperature of enzyme kinetics in unwarmed (at 20°C) and warmed soils (30°C). Error bars indicate standard errors (*n* = 3).

SUPPLEMENTARY FIGURE S3 Relationship of warming-induced declines in soil enzyme activities (measured by *R*_30/20_, the ratio of levels at 30–20°C) to that in total PLFA at the late incubate stage. Soils were amended with or without secondary rice litter inputs. The *R*_30/20_ for enzyme activities was averaged over substrate concentrations of 200–600 μm (Figure 4). Two exponential equations were fitted for LAP and NAG enzymes. The correlation for BG was insignificant due to two outliers (within the dashed area).
