# Supplementary material for: Contrasting effects of maize litter and litter-derived biochar on the temperature sensitivity of paddy soil organic matter decomposition
Source: Front Microbiol. 2022 Sep 29;13:1008744. doi: 10.3389/fmicb.2022.1008744 (PMC9557949; doi:10.3389/fmicb.2022.1008744)

Table S1 PLFA markers used to identify microbial groups and to indicate the influences of temperature stress.

| Taxonomic groups | PLFA markers |
| --- | --- |
| Gram-negative bacteria | 3OH10:0, 14:1ω5, 15:1ω6, 15:1ω7, 16:1ω7, 16:1 ω9, cy17:0ω7, 17:1ω8, 18:1ω5, 18:1ω7, cy19:0ω7, 19:1ω7, 19:1ω8, 20:1ω8, 20:1ω9, 21:1ω3, 21:1ω6, 21:1ω8, 22:1ω3 |
| Gram-positive bacteria | a11:0, a12:0, a13:0, i13:0 iso, a14:0, i14:0, a15:0, i15:0, i15:1ω6, a16:0, i16:0, a17:0, i17:0, i17:1ω9, i18:0, a19:0 |
| Actinomycetes | 10Me16:0, 10Me17:0, 10Me17:1ω7c, 10Me18:0, 10Me18:1ω7, 10Me20:0 |
| Fungi | 18:2ω6, 18:1ω9 |
| Temperature stress indicators |  |
| Cy/Pre ratio | (cy17:0+cy19:0) /(16:1ω7+18:1ω7) |
| PLFA unsaturation | ∑(Unsaturated PLFA amount × number of their double bonds)/(total PLFA) |

Table S2 Contents of phospholipid fatty acids (PLFAs) for various microbial groups at the early stage of soil incubation. Values are presented as Mean ± standard error (n=3). G^-^ and G^+^ bacteria refer to Gram-negative and Gram-positive bacteria, respectively. Different lowercase letters in a column indicate significant differences between treatments.

|  | Total PLFA (nmol g^-1^) | PLFA for microbial groups (nmol g^-1^) | | | | |  | Fungi/bacteria |
| --- | --- | --- | --- | --- | --- | --- | --- | --- |
|  |  | G^-^ bacteria | G^+^ bacteria | Actinomycetes | Fungi | Other |  |  |
| Control | 85.3±4.3b | 26.4±0.6b | 16.5±0.4bc | 12.3±0.2b | 3.0±0.6b | 30.4±3.1b |  | 0.07±0.01b |
| Litter | 220±6.6a | 63.7±1.5a | 34.8±1.9a | 16.6±0.3a | 33.3±1.2a | 717±2.1a |  | 0.34±0.01a |
| BC400 | 84.2±0.6b | 25.5±0.7bc | 18.8±1.2b | 10.6±0.4c | 2.4±0.04b | 27.0±0.7b |  | 0.05±0.001b |
| BC650 | 73.7±0.8b | 23.3±0.1c | 13.5±0.6c | 9.7±0.08c | 2.2±0.02b | 25.0±0.2b |  | 0.06±0.001b |

**Figure S1**


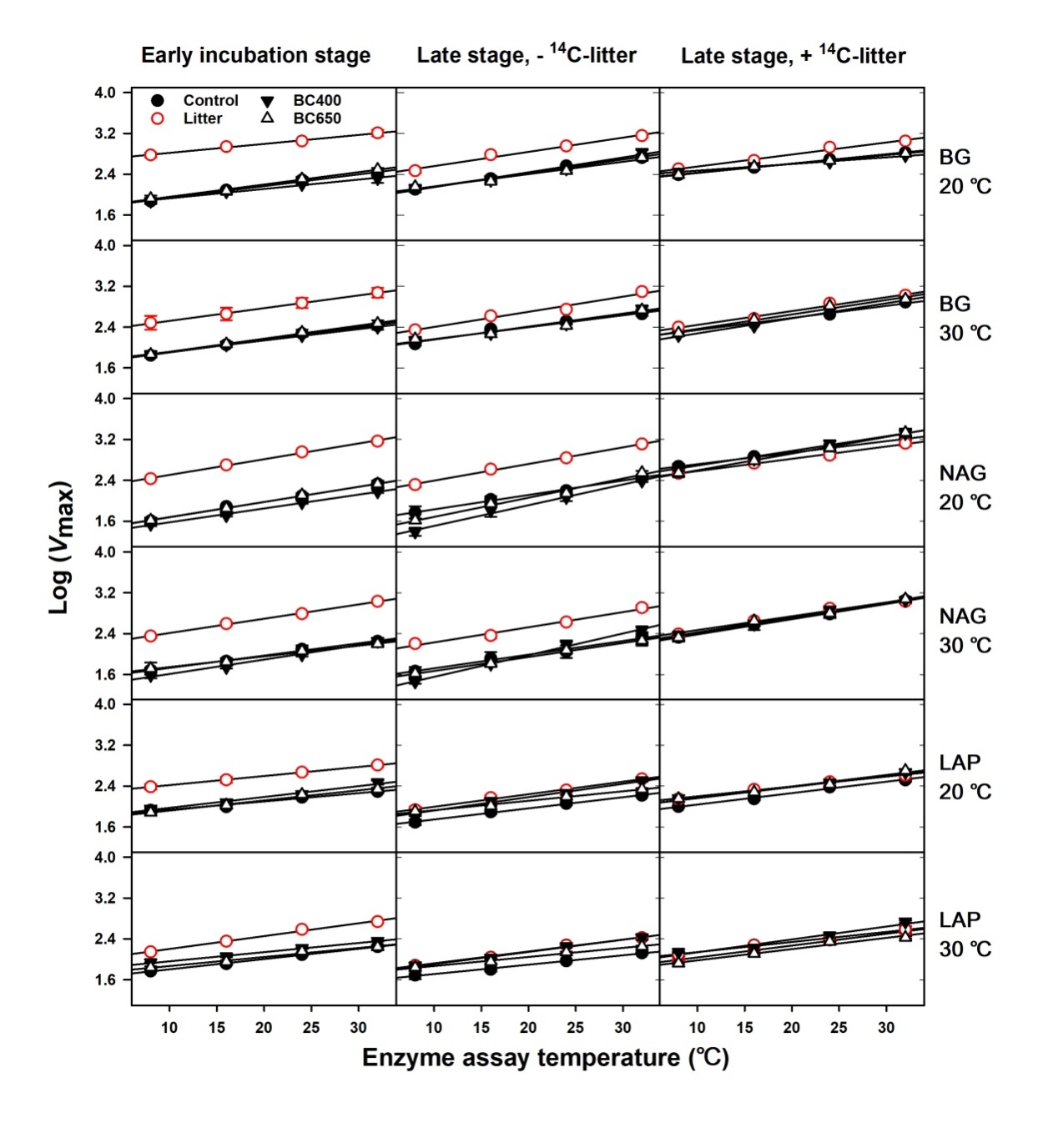


**Figure S2**


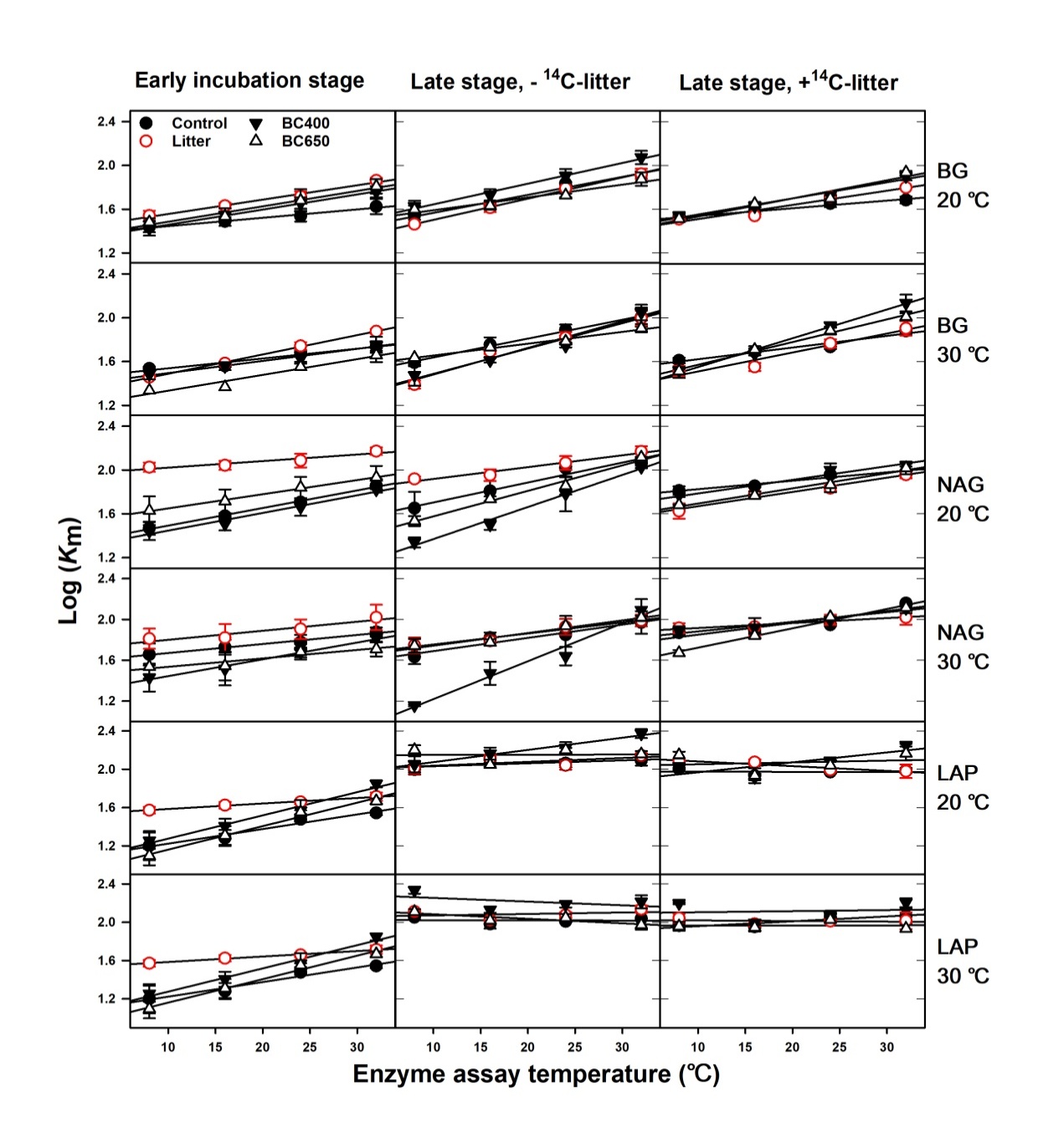


**Figure S3**


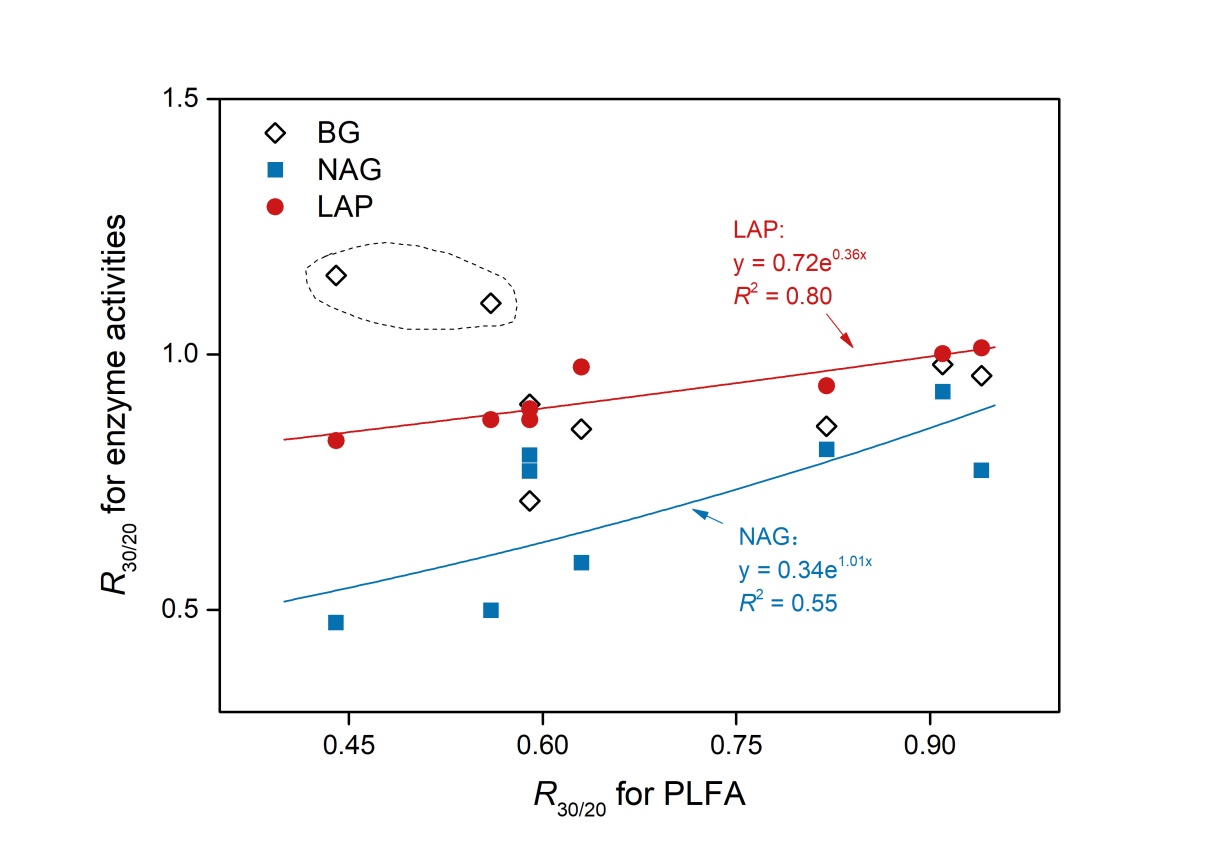

Supplement: SUPPLEMENTARY FIGURE S1 — Relationship between log(Vmax) and assay temperature of enzyme kinetics in unwarmed (at 20°C) and warmed soils (30°C). Error bars indicate standard errors (n = 3). [file Data_Sheet_1.docx]
